# Spliceosomal vulnerability of *MYCN*-amplified neuroblastoma is contingent on PRMT5-mediated regulation of epitranscriptomic and metabolomic pathways

**DOI:** 10.1016/j.canlet.2024.217263

**Published:** 2024-09-21

**Authors:** Jodie Bojko, Madhu Kollareddy, Marianna Szemes, Jacob Bellamy, Evon Poon, Ahmad Moukachar, Danny Legge, Emma E. Vincent, Nicholas Jones, Sally Malik, Alexander Greenhough, Alex Paterson, Ji Hyun Park, Kelli Gallacher, Louis Chesler, Karim Malik

**Affiliations:** a Cancer Epigenetics Laboratory, School of Cellular and Molecular Medicine, University of Bristol, Bristol, UK; b Division of Clinical Studies, The Institute of Cancer Research, London, UK; c Translational Health Sciences, Bristol Medical School, University of Bristol, Bristol, UK; d Institute of Life Science, Swansea University Medical School, Swansea University, Swansea, SA2 8PP, UK; e College of Health, Science and Society, University of the West of England, Bristol, BS16 1QY, UK; f Insilico Consulting ltd, Wapping Wharf, Bristol, England, UK

## Abstract

Approximately 50 % of poor prognosis neuroblastomas arise due to *MYCN* over-expression. We previously demonstrated that MYCN and PRMT5 proteins interact and PRMT5 knockdown led to apoptosis of *MYCN-*amplified (MNA) neuroblastoma. Here we evaluate the highly selective first-in-class PRMT5 inhibitor GSK3203591 and its *in vivo* analogue GSK3326593 as targeted therapeutics for MNA neuroblastoma. Cell-line analyses show MYCN-dependent growth inhibition and apoptosis, with approximately 200-fold greater sensitivity of MNA neuroblastoma lines. RNA sequencing of three MNA neuroblastoma lines treated with GSK3203591 reveal deregulated MYCN transcriptional programmes and altered mRNA splicing, converging on key regulatory pathways such as DNA damage response, epitranscriptomics and cellular metabolism. Stable isotope labelling experiments in the same cell lines demonstrate that glutamine metabolism is impeded following GSK3203591 treatment, linking with disruption of the MLX/Mondo nutrient sensors via intron retention of *MLX* mRNA. Interestingly, glutaminase (GLS) protein decreases after GSK3203591 treatment despite unchanged transcript levels. We demonstrate that the RNA methyltransferase METTL3 and cognate reader YTHDF3 proteins are lowered following their mRNAs undergoing GSK3203591-induced splicing alterations, indicating epitranscriptomic regulation of GLS; accordingly, we observe decreases of GLS mRNA m6A methylation following GSK3203591 treatment, and decreased GLS protein following YTHDF3 knockdown. *In vivo* efficacy of GSK3326593 is confirmed by increased survival of *Th-MYCN* mice, with drug treatment triggering splicing events and protein decreases consistent with *in vitro* data. Together our study demonstrates the PRMT5-dependent spliceosomal vulnerability of MNA neuroblastoma and identifies the epitranscriptome and glutamine metabolism as critical determinants of this sensitivity.

## 1 Introduction

Neuroblastoma is one of the most common solid tumours of childhood, and approximately 50 % of neuroblastoma patients have a high-risk clinical phenotype with very poor prognosis, specifically a long-term survival rate of less than 40 % [1]. The earliest defined driver of poor prognosis neuroblastoma is gene amplification of the *MYCN* proto-oncogene which encodes a transcription factor of the myc-family [2]. *MYCN*-amplified neuroblastoma (MNA neuroblastoma) represents about half of poor prognosis neuroblastoma, with the remainder attributable to enhancer alterations leading to over-expression of *TERT* [3] or *MYC* [4].

Regrettably, these oncogenic drivers are not amenable to targeted therapies, underlining the crucial need for identifying druggable synthetic lethal or collateral vulnerabilities for efficacious therapies. Previous work in our laboratory demonstrated that MNA neuroblastoma cell survival was dependent on protein arginine methyltransferase 5 (PRMT5), with knockdown of PRMT5 resulting in MNA neuroblastoma cell line apoptosis [5]. PRMT5 is one of two “type II” arginine methyl-transferases, the other being PRMT9. Type II PRMTs catalyse symmetric arginine dimethylation (SDMA), in contrast to asymmetric arginine dimethylation (type I PRMTs) or monomethylarginine (type III PRMTs). PRMT5 and PRMT9 are not functionally redundant and exhibit very distinct substrate preferences [6]. PRMT5 has pleiotropic functions and exerts oncogenicity by different mechanisms in many cancers [6,7], by functioning as a writer of symmetric dimethylation of the histone 3 at arginine 2 (H3R2me2s), arginine 8 (H3R8me2s) and histone 4 arginine 3 (H4R3me2s) marks associated with epigenetic silencing, exemplified by PRMT5 maintenance of breast cancer stem cells by epigenetically regulating *FOXP1* [8]. Non-histone proteins are also substrates for PRMT5, with glycine-arginine rich (GAR) motifs being the preferred but not exclusive methylation sites and methylation by PRMT5 can alter the stability and activity of key transcription factors including p53 [9], E2F-1 [10,11], and MYCN in neuroblastoma [5]. PRMT5 can also methylate signalling proteins such as RAF proteins [12] and Akt [13,14].

Crucially, PRMT5 is a vital regulator of constitutive and alternative mRNA splicing (AS) via arginine methylation of Sm proteins, small nuclear ribonucleoproteins essential for assembly of snRNP core particles in the spliceosome [15,16]. Co-opting of PRMT5 and spliceosomal regulation has been shown to be essential for lymphomagenesis in an Eμ-myc driven mouse model [17], underlining the critical role of alternative splicing in cancer.

Given the evidence for PRMT5 representing a collateral vulnerability for the MYC-family proteins from our lab and others [5,17] we reasoned that pharmaceutical inhibition of PRMT5 may represent a promising targeted therapy for high-risk neuroblastoma. In this study, we report assessment of the substrate competitive PRMT5 inhibitor structural analogues GSK3203591 and GSK3326593 *in vitro* and *in vivo* respectively. These highly selective and potent inhibitors were discovered by GlaxoSmithKline and display >4000-fold selectivity over other methyltransferases, negating possible off-target effects [18,19]. As our previous immunohistochemical analyses of primary tumours had demonstrated very high nuclear PRMT5 in poor prognosis MNA neuroblastoma, in contrast to predominantly cytoplasmic PRMT5 in other neuroblastomas [5], we focused on nuclear deregulatory events. Our work demonstrates that PRMT5 activity is integral to regulation of transcriptional and alternative splicing programmes in MNA neuroblastoma, functioning to regulate key survival and fitness programmes, including cellular metabolism, DNA repair and epitranscriptome modulation.

## 2 Results

### 2.1 MYCN amplified neuroblastoma cell lines are preferentially sensitive to pharmaceutical inhibition of PRMT5

**Fig. 1:**
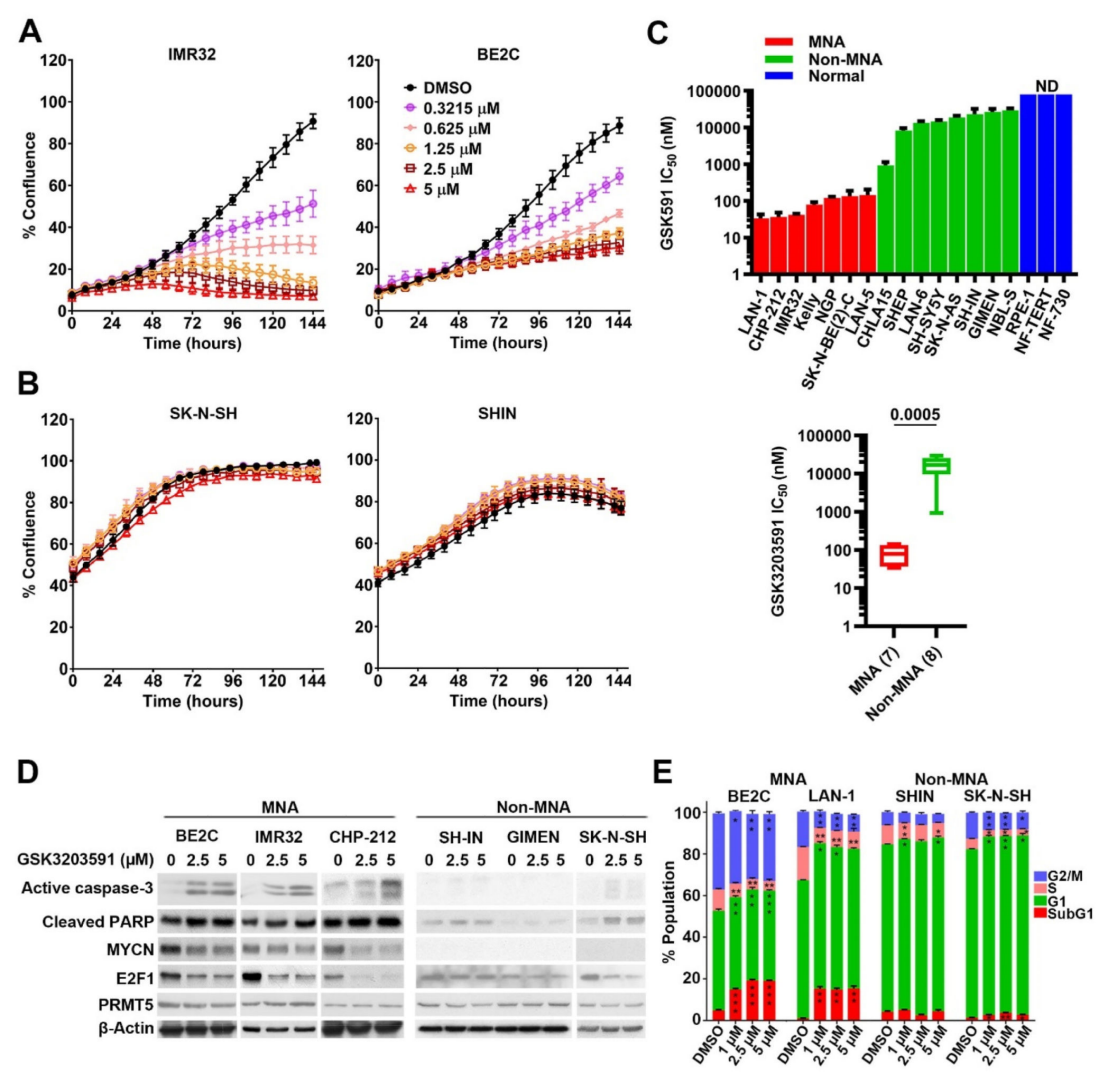
MYCN amplified cells are preferentially sensitive to PRMT5 inhibition by GSK3203591. **[A]** Incucyte Zoom live cell imaging of two MYCN amplified (MNA) neuroblastoma lines demonstrating dose-dependent growth inhibition following GSK3203591 treatment (n = 3, mean ± SD). **[B]** Imaging, as above, of two non-MNA neuroblastoma lines demonstrating no significant growth inhibition following GSK3203591 treatment (n = 3, mean ± SD) **[C]**. *(Upper panel)* GSK3203591 IC_50_ values for 7 MNA, 8 non-MNA and 3 non-cancerous cell lines (n = 3; mean ± SD, ND: not determined). *(Lower panel)* Box plot showing statistically significant mean IC_50_ ± SEM values of MNA vs non-MNA (*** = p < 0.0005, unpaired *t*-test). **[D]** Immunoblot of apoptotic markers, PRMT5, MYCN and E2F1 in cell extracts prepared from MNA (BE2C, IMR32, CHP-212) and non-MNA (SH-IN, GIMEN, SK-N-SH) neuroblastoma cell lines (n = 2). **[E]** Flow cytometry-based cell cycle analysis of two MNA (BE2C and LAN-1) and two non-MNA (SH-IN and SK-N-SH) cell lines, demonstrating increased sub-G1 apoptotic population and redistribution of the cell cycle (n = 3, mean ± SD, * = p < 0.05, ** = p < 0.005, *** = p < 0.0005, student’s one-tailed *t*-test).

We sought to further develop our genetic interference data demonstrating PRMT5 requirement in MNA neuroblastoma [5] by evaluating the highly selective, first-in-class PRMT5 inhibitor GSK3203591 on a panel of neuroblastoma cell lines. Initially we investigated growth inhibition using live-cell imaging of two MNA and two non-MNA neuroblastoma lines. Whereas the MNA lines IMR32 and SK-N-BE(2)-C (herein referred to as BE2C) demonstrated dose-dependent growth inhibition, the non-MNA lines SK-N-SH and SH-IN did not (Fig. 1A–B). We extended this analysis by determining IC_50_s for GSK3203591 in a panel of 14 neuroblastoma lines, together with three non-cancer lines RPE-1, NF-730 and NF-TERT. This verified that MNA-cell lines are >200 times more sensitive to the effects of PRMT5 inhibition compared to non-MNA lines, with mean IC_50_ values of 84 nM for MNA lines (range 33 nM–144 nM) and 19.15 μM (range 8.3 μM–29.4 μM) for non-MNA lines (Fig. 1C). The non-cancer lines did not display IC_50_s in the concentration range 625 nM-80 μM used for non-MNA and non-cancer lines.

We next examined GSK3203591 effects on potential molecular targets and markers of apoptosis. Cleaved caspase-3 and cleaved PARP increased with GSK3203591 treatment in three MNA-lines, but barely changed in non-MNA cell lines (Fig. 1D), although profound apoptosis was not observed with the treatment parameters. MYCN and E2F1 proteins were decreased, whereas PRMT5 levels were unchanged. All treatments had decreased symmetrical dimethyl arginine (SDMA) and not affected asymmetrical dimethyl arginine (ADMA), confirming on target drug specificity (Supplementary Fig. 1). We further assessed apoptosis using cell cycle analyses to quantify the increased sub-G1 apoptotic population, confirming significant changes in the sub-G1 fraction on two MNA neuroblastoma lines, and minimal changes in the non-MNA lines, consistent with the immunoblots (Fig. 1E). We also observed G1-phase increases and decreased cells in S-phase as previously shown [19]. Together our analyses identify a *MYCN* amplification dependent sensitivity of neuroblastoma cell-lines to GSK3203591.

### 2.2 MYCN expression sensitizes cells to GSK3203591

**Fig. 2:**
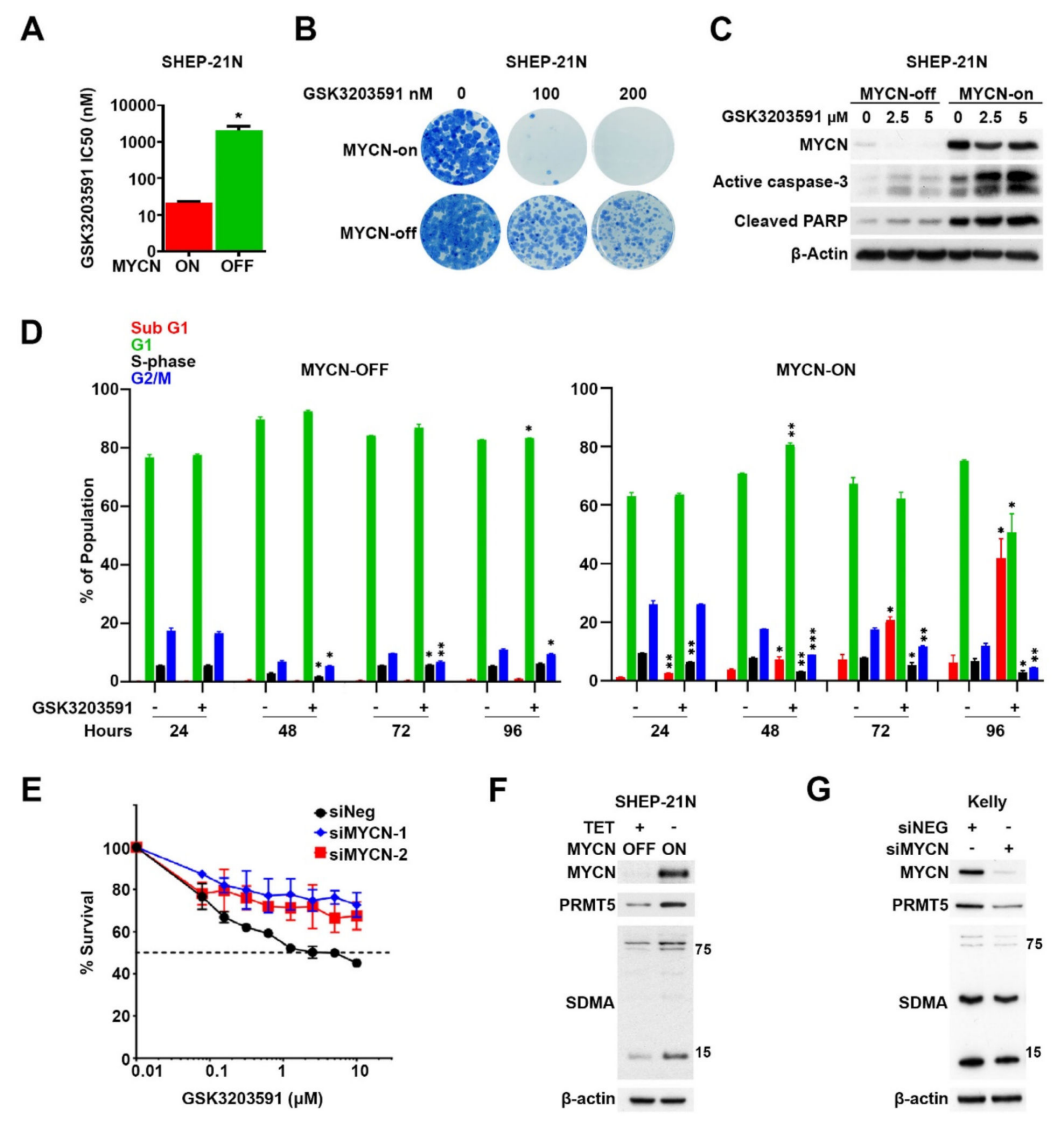
MYCN expression sensitizes neuroblastoma cells to PRMT5 inhibition by GSK3203591. **[A]**. The isogenic neuroblastoma line SHEP-21N, with tetra-cycline regulable MYCN expression, shows increased GSK3203591 sensitivity when MYCN is switched on (n = 3, mean ± SD, * = p < 0.05, unpaired *t*-test). **[B]** Clonogenic assay with SHEP-21N cells demonstrating MYCN-dependency for GSK3203591-mediated growth inhibition (n = 2). **[C]** Immunoblot analysis of cell extracts from SHEP-21N cells treated with GSK3203591 plus with (MYCN off) and without (MYCN on) tetracycline showing MYCN-dependent increases in apoptotic markers (n = 2). **[D]** Flow cytometry cell cycle analysis by DNA content (propidium iodide) in SHEP-21N cells treated with 5 μM GSK3203591 or DMSO equivalent incubated with (MYCN off) and without (MYCN on) tetracycline for 24–96 h time course (n = 2, mean ± SD, * = p < 0.05, ** = p < 0.005, *** = p < 0.0005, unpaired *t*-test). **[E]** Cell survival assay (MTT) of BE2C cells transfected with two different siRNA’s targeting MYCN or siNEG and incubated with increasing concentrations of GSK3203591 for 96 h (n = 3, mean ± SD). **[F]** Immunoblot analysis of cell extracts from SHEP-21N cells incubated with (MYCN off) and without (MYCN on) tetracycline showing MYCN-dependent increases in PRMT5 and SDMA (n = 3). **[G]** Immunoblot of cell extracts from Kelly cells transfected with siRNA targeting MYCN showing MYCN dependent PRMT5 and SDMA expression (n = 3).

We next assessed the potential MYCN dependency of GSK3203591 sensitivity using isogenic SHEP-21N cells with tetracycline regulable MYCN [20]. Proliferation assays revealed approximately 90-fold increased sensitivity with MYCN induced, i.e. an IC_50_ of 22.6 ± 1.93 nM with MYCN induced and at 2.07 ± 1.2 μM when MYCN is off (Fig. 2A). Reflecting this, clonogenic assays demonstrated reduced colony formation in MYCN-on cells (Fig. 2B). As the increased sensitivity to GSK3203591 could be attributable to the faster proliferation rate of MYCN-on cells, we also tested etoposide as a control. However, etoposide sensitivity did not segregate with MYCN expression status (Supplementary Fig. 2A), further indicating that PRMT5 inhibition effects are highly specific to MYCN status. In line with this, apoptotic markers were more markedly increased in MYCN-on cells (Fig. 2C). This trend was also apparent in cell cycle analyses revealing a significant increase in sub-G1 cells in MYCN-on cells, and not in MYCN-off cells. The cell cycle distribution analysis also indicated a smaller fraction of cells in S-phase in MYCN-on cells after 24 h treatment (Fig. 2D). We next used the more sensitive technique of BrdU incorporation into nascent DNA combined with propidium iodide stain to accurately measure the amount of cells in S-phase, and we demonstrate significantly reduced S-phase populations denoting decreased DNA replication following GSK3203591 treatment (Supplementary Fig. 2D).

To further verify the MYCN-dependent sensitivity, we knocked down endogenous MYCN in BE2C cells and concurrently treated with GSK3203591 (Fig. 2E and Supplementary Fig. 2B). MYCN knockdowns increased resistance to GSK3203591, suggesting MYCN expression is essential for PRMT5 inhibition effects. As a control we also tested doxorubicin, but doxorubicin potency was not affected by MYCN knockdown (Supplementary Fig. 2C). PRMT5 has been shown to be a c-myc target [17] and MYCN has been shown to bind the PRMT5 promoter [21], prompting us to evaluate MYCN effects on PRMT5 protein and symmetrical dimethylarginine (SDMA) using the SHEP-21N system. Induction of MYCN protein led to an increase in both PRMT5 and the SDMA mark (Fig. 2F), and conversely, knockdown of MYCN in Kelly cells led to a decrease in PRMT5 and SDMA (Fig. 2G). This is consistent with our previous demonstration of higher PRMT5 in MNA cell-lines [5], a pattern mirrored by the obligate methylosome PRMT5 interacting protein WDR77/MEP50, previously shown to be a direct target of MYCN [22] (Supplementary Figs. 2E–F).

Taken together, these studies verify that expression of MYCN protein in neuroblastoma cells is a major determinant of GSK3203591 sensitivity, and further validate a MYCN-PRMT5 axis crucial to survival and proliferation of neuroblastoma as suggested previously using PRMT5 knockdowns [5].

### 2.3 PRMT5 inhibition leads to widespread gene expression changes, including MYCN-regulated genesets

**Fig. 3:**
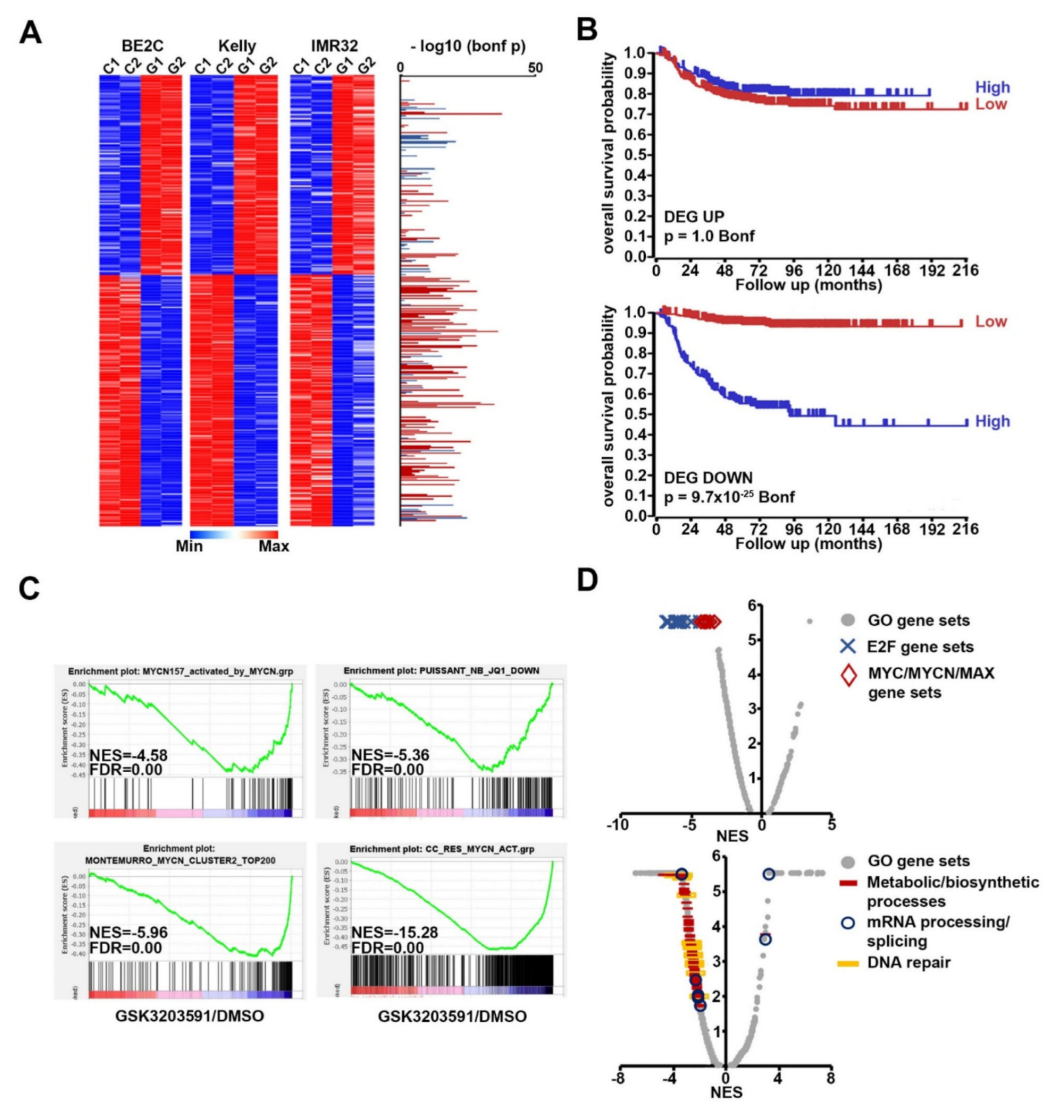
RNA sequencing of GSK3203591-treated MNA neuroblastoma lines reveals down-regulation of MYCN-activated genes. **[A]** Heatmaps of 315 differentially expressed genes (DEG, adjusted p < 0.05, ± 30 % change) shared by BE2C, Kelly and IMR32 cell-lines following GSK3203591 treatment (C, control(DMSO); G, GSK3203591). Associations with prognosis for all genes is indicated alongside (right), bars indicate high expression is associated with poor prognosis (red) or good prognosis (blue). Bonferroni-corrected p-values were calculated on the R2 Genomics Analysis and Visualization Platform (http://r2.amc.nl) using the SEQC dataset containing gene expression data from 498 neuroblastoma patients. RNAseq was performed using n = 2 biological replicates. **[B]** Kaplan Meier plots of gene signatures (metagenes) from genes upregulated following PRMT5 inhibition (DEG UP, top) or down-regulated (DEG DOWN, bottom) against overall survival. Note that high expression of DEG DOWN genes is strongly associated with poor survival. **[C]** GSEA analysis of GSK3203591-treated IMR32 cells demonstrating strong inhibition of MYCN-dependent gene sets. **[D]** Global summary of GSEA plots showing repression of MYC/MYCN and E2F gene sets (top) and other frequently altered gene ontology GSEAs affected by PRMT5 inhibition (bottom). NES, normalised enrichment score; FDR, false discovery rate.

To understand how GSK3203591 exerts growth inhibitory effects on MNA cell lines, we next sought to examine how PRMT5 inhibition may alter gene expression programmes in MNA lines. IMR32, Kelly and BE2C were treated with GSK3203591 and transcriptomes analysed by RNA sequencing. We found 315 differentially expressed genes (DEGs, adjusted p < 0.05, ± 30 % change) common to all 3 cell lines (Supplementary Fig. 3A), and focused further investigation to this overlap set to find mechanistic determinants underscored by *MYCN* amplification and over-expression (Fig. 3A). This set included 138 genes upregulated after GSK3203591 treatment and 177 genes downregulated genes (Supplementary File 1 – Table 1). A panel of 29 DEGs was validated by qRT-PCR in all 3 cell lines (Supplementary Fig. 3B). Interestingly, scanning of expression levels and correlation with prognosis using the R2: Genomics Analysis and Visualization Platform (http://r2.amc.nl) suggested that an upregulated DEGs signature (DEG UP) did not significantly correlate with overall survival probability. Strikingly, the DEG DOWN signature showed a dramatic correlation with poor survival (p = 9.7x10^-25^) (Fig. 3B). Thus, high expression of the majority of genes from the DEG-DOWN signature associate with very poor prognosis in primary neuroblastoma, and PRMT5 inhibition lowers their levels in MNA neuroblastoma. These signatures suggest that PRMT5-mediated epigenetic silencing is unlikely to be the major determinant of MNA neuroblastoma sensitivity to GSK3203591. Conversely poor prognosis genes, potentially highly expressed because of MYCN-mediated trans-activation, are mediators of increased survival and fitness of MNA neuroblastoma cells. The expression of these genes and their pathways are compromised by PRMT5 inhibition.

We next conducted gene set enrichment analysis (GSEAs) to identify key pathways altered following PRMT5 inhibition. MYCN-activated genesets were strongly repressed in IMR32 cells (Fig. 3C), as well as in Kelly and BE2C cell lines (Supplementary Fig. 3C). Volcano plots of GSEAs indicated frequent decreases in MYC/MYCN/MAX and E2F genesets, the latter likely reflecting the regulation of E2F transcription factors by MYCN [23–25]. Other notable changes (predominantly decreases) were observed in metabolic/biosynthetic process and mRNA processing, splicing and DNA repair (Fig. 3D and Supplementary Fig. 3D). Despite the strong association of DEGs with MYCN, the 29 DEGs validated by qRT-PCR showed similar expression changes in GSK3203591-treated non-MNA neuroblastoma lines (Supplementary Fig. 3E), likely due to the overlapping activities of c-MYC and MYCN. Nevertheless, our RNAseq analyses show that GSK3203591 treatment suppresses transcription of genes involved in fitness, survival and proliferation pathways.

### 2.4 PRMT5 inhibition leads to multiple alternative splicing events in MNA neuroblastoma

**Fig. 4:**
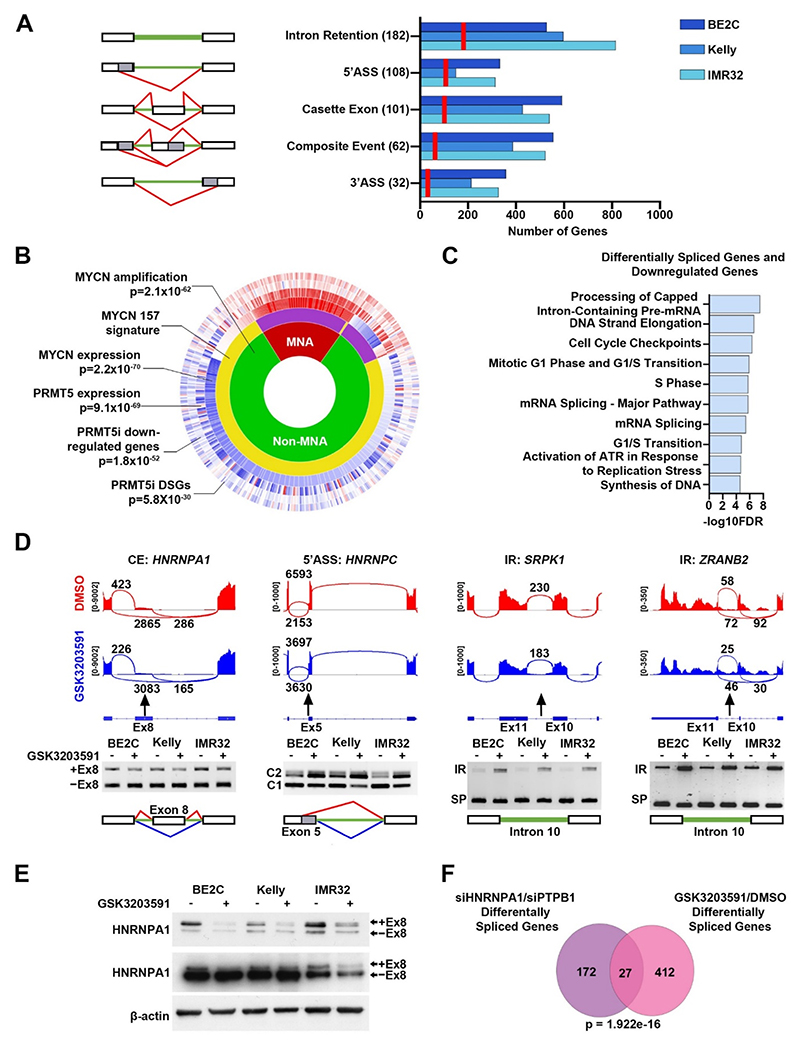
PRMT5 inhibition in MYCN-amplified neuroblastoma cell lines leads to consistent and widespread alternative splicing. **[A]** Numerical summary of alternative splicing events occurring in genes following PRMT5 inhibition in 3 MNA cell lines (BE2C, Kelly, IMR32) (right). Red lines on bar graph indicate the number of shared genes with alternative splicing events in the 3 cell lines, exact number displayed in brackets. Schematic (left) shows introns (green), exons (white) and splicing (red) (3′ASS, 3′alternative splice site; 5′ASS, 5′alternative splice site). **[B]** Sunburst plot generated in R2 (http://r2.amc.nl) showing, from inner to outer, correlation of *MYCN* amplification status (MNA/non-MNA), the MYCN-157 prognostic signature, *MYCN* transcription levels, *PRMT5* transcription levels, differentially expressed downregulated genes following PRMT5 inhibition (PRMT5i), and differentially spliced genes (DSGs) following PRMT5i. Overlap probabilities are shown relative to the MYCN-157 signature. **[C]** Top 10 significantly enriched Reactome pathways in a combined list of differentially spliced genes and downregulated differentially expressed genes. **[D]** Representative sashimi plots (top) and end-point PCR validation (bottom, n = 3) of alternative splicing events occurring in genes that function in RNA splicing in BE2C, Kelly and IMR32 cells after GSK3203591 (IR, intron retention; SP, spliced product; CE, cassette exon; 5′ASS, 5′ alternative splice site; lines indicate splicing before (red) and after (blue) GSK3203591). **[E]** Immunoblot of cell extracts from BE2C, Kelly, IMR32 cells treated with GSK3203591 or DMSO equivalent (96h) showing decreased protein expression of hnRNPA1 (hnRNPA1B custom antibody preferentially targeting the + exon 8 isoform (top) and commercial antibody detecting total hnRNPA1 (bottom) (n = 3). **[F]** Venn diagram of differentially spliced genes in neuroblastoma cells transfected with siRNA targeting HNRNPA1/PTPB1 and differentially spliced genes shared by the 3 neuroblastoma cell lines following GSK3203591 treatment in this study.

As PRMT5 is a key regulator of alternative splicing, further analysis of RNAseq data was conducted to identify differentially spliced genes (DSGs). Altogether 439 genes had at least 1 alternative splicing event shared between the 3 cell lines. These comprised of 32 genes with 3′-alternative splice sites (3′-ASS), 108 with 5′-alternative splice sites (5′-ASS), 101 with alternative cassette exons (CE), 62 with composite events (CompE) and 182 genes with ≥1 intron retention (IR) (Fig. 4A, Supplementary File 1 – Table 2). We compared the potential association of DEGs and DSGs with neuroblastoma prognosis, specifically with the MYCN-157 gene signature that was found to have greater prognostic power than *MYCN* amplification status [22]. As shown in Fig. 4B, there are highly significant overlaps between DEGs, DSGs, PRMT5 expression versus the MYCN-157 signature, indicating that the genes which showed altered expression following PRMT5 inhibition, together with PRMT5, had significantly higher expression in MNA neuroblastoma and were co-expressed with the MYCN157 prognostic signature in the SEQC neuroblastoma dataset of 498 tumours. This shows that the PRMT5 regulated DEGs and DSGs strongly overlap with genes linked to poor prognosis. Reactome analysis highlighted RNA splicing/processing, cellular metabolism, and DNA repair as over-represented categories amongst downregulated DEGs and DSGs (Fig. 4C). We first validated RNA splicing/processing hits identified amongst our DSGs; examples identified in our data include skipping of exon 8 in *HNRNPA1*, 5′-ASS of *HNRNPC*, and intron retention of *SRPK1* and *ZRANB2*. To our knowledge, these AS events have not been reported as being regulated by PRMT5 before (Fig. 4D). Protein level changes of hnRNPA1 following GSK3203591 treatment were also confirmed in MNA neuroblastoma cell-lines (Fig. 4E). Interestingly, hnRNPA1 was previously demonstrated to be a MYCN target gene mediating AS in neuroblastoma [26], and a comparison of our DSGs with DSGs reported in RNAseq of hnRNPA1/PTBP1 knockdowns [26] demonstrated a significant overlap (Fig. 4F). Several other genes encoding splicing and RNA metabolism regulators, including *PAPOLA, HNRNPD, HNRNPH1* and *PRPF3*, were also validated at the RNA level (Supplementary Fig. 4A).

Two recent studies demonstrated that targeting of the splicing regulator RBM39 using indisulam represents a novel therapeutic option for neuroblastoma [27,28]. We therefore examined the extent to which our DSGs overlapped with DSGs following RBM39 knockdown in BE2C [27]. Whilst a strikingly significant overlap was apparent between the two genesets, we observed no GSK3203591 effects on RBM39 protein levels (Supplementary Figs. 4B–C).

**Fig. 5:**
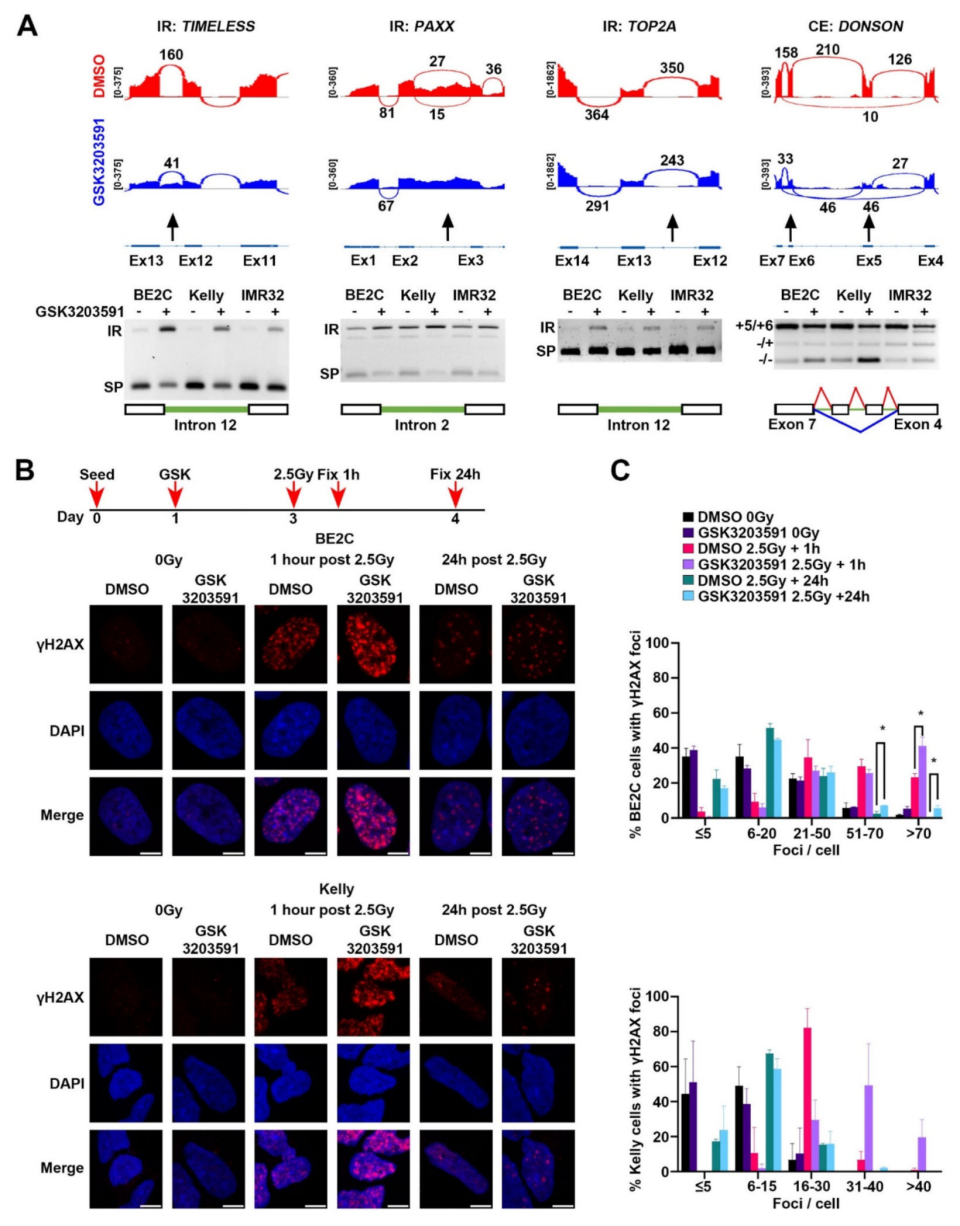
GSK3203591 induced alternative splicing of DNA repair factors and increased irradiation induced DNA damage. **[A]** Representative sashimi plots (top) and end-point PCR validation (bottom, n = 3) of alternative splicing events occurring in genes that function in DNA damage response and repair (*TIMELESS, PAXX, TOP2A, DONSON*) in BE2C, Kelly and IMR32 cells treated with GSK3203591 or DMSO equivalent (96h) (IR, intron retention; SP, spliced product; CE, cassette exon; lines indicate splicing before (red) and after (blue) GSK3203591). **[B]** Representative confocal microscopy images (Z-projections) of γH2AX (red) in BE2C (top, n = 3) and Kelly (bottom, n = 2) cells treated with GSK3203591 and irradiated (nuclear counterstain Hoechst, blue; 63× magnification, scale bar 5 μm). **[C]** Quantification of γH2AX foci per nucleus represented in B for BE2C (top, ≥125 cells counted from 3 experiments) and Kelly (bottom, ≥91 cells counted from 2 experiments) (mean ± SEM, unpaired *t*-test, * = p < 0.05).

PRMT5 inhibition has previously been reported to regulate the DNA damage response via alternative splicing of *KAT5* exon 5 [29]. We also observed the AS of *KAT5* in our data (Supplementary Fig. 4D) and in addition, we identified novel intron retention events in DNA repair genes *TIMELESS, PAXX* and *TOP2A*, as well as cassette exon splicing in *DONSON* (Fig. 5A). We assessed the effect of PRMT5 inhibition on DNA damage repair in BE2C and Kelly cell lines by inducing double stranded DNA breaks by gamma irradiation after 48 h GSK3203591 treatment. Increased DNA damage foci were observed in GSK3203591 treated BE2C and Kelly cells 1-h post irradiation and DNA damage foci persisted 24-h post irradiation in GSK3203591 treated BE2C cells (Fig. 5B–C). These results show GSK3203591 impairs the DNA damage response and repair of double stranded DNA breaks in neuroblastoma.

Together our AS analysis strongly supports deregulation of splicing programmes by PRMT5 as an important factor in neuroblastoma growth and survival.

### 2.5 PRMT5 inhibition impacts neuroblastoma cellular metabolism

**Fig. 6:**
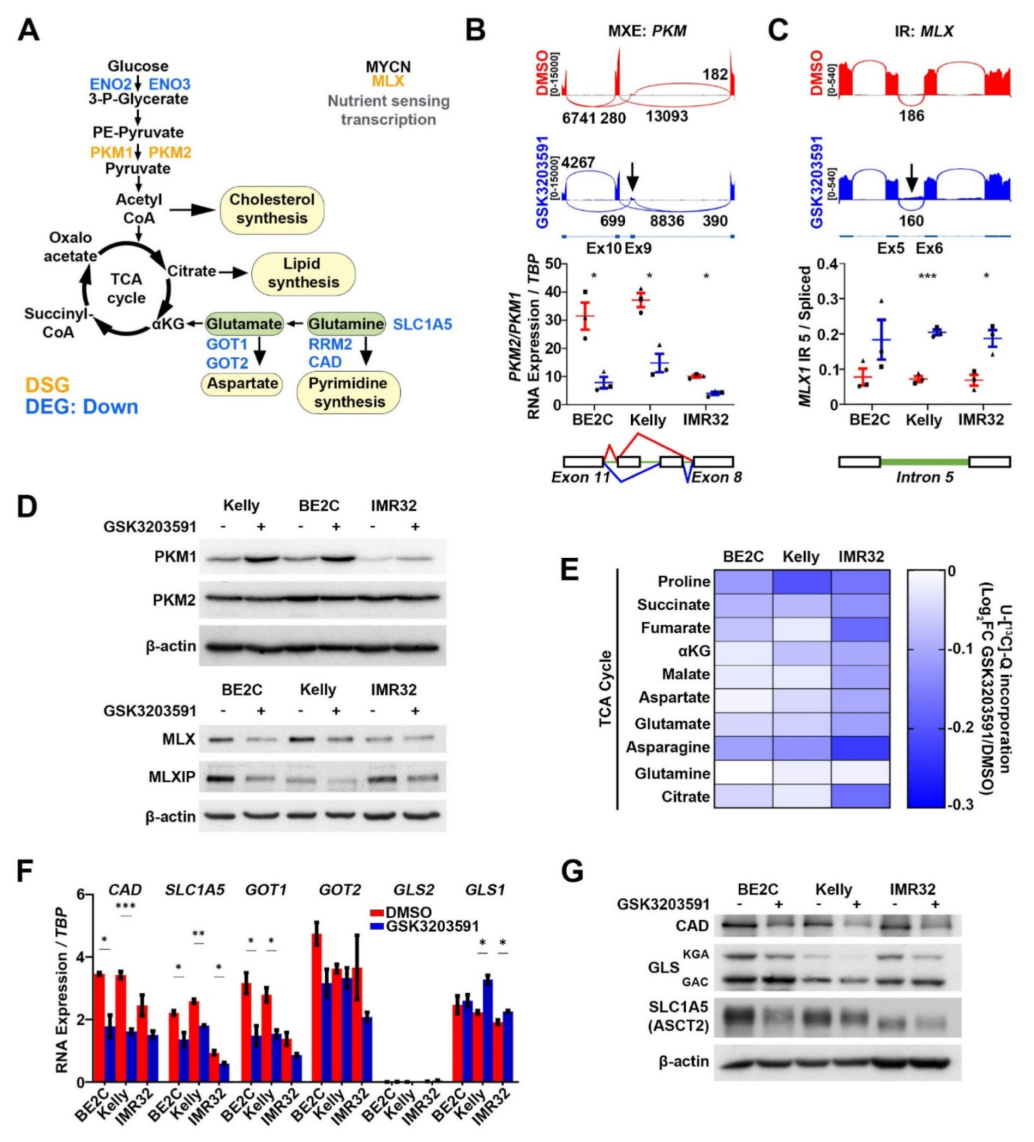
PRMT5 inhibition by GSK3203591 impacts cellular fitness by downregulating glutamine metabolism regulators. **[A]** Schematic diagram of glucose and glutamine metabolism highlighting differentially spliced genes (orange, DSG) and differentially expressed downregulated genes (blue, DEG Down) after GSK3203591 treatment. **[B]** Representative sashimi plot (top) and RT-qPCR validation (bottom) of PKM isoform expression (n = 3, mean ± SEM, unpaired *t*-test, * = p < 0.05, MXE = mutually exclusive exon). **[C]** Representative sashimi plot (top) and RT-qPCR validation (bottom) of intron retention of intron 5 in the *MLX* transcript after GSK3203591 treatment (n = 3, mean ± SEM, unpaired *t*-test, * = p < 0.05, *** = p < 0.0005, IR = intron retention) **[D]** Immunoblot of cell extracts from MNA cells treated with GSK3203591 showing PKM isoform switching from PKM2 to PKM1 (top) and downregulation of MLX and MLXIP expression (bottom) (n = 3). **[E]** Heatmap summarizing decreased glutamine incorporation into TCA cycle intermediates after GSK3203591 relative to control (data derived from n = 3 stable isotope labelling experiments). **[F]** RT-qPCR showing decreased expression of glutamine metabolism genes in MNA cell lines after GSK3203591 (n = 3, mean ± SEM, unpaired *t*-test * = p < 0.05, ** = p < 0.005, *** = p < 0.0005). **[G]** Immunoblot showing decreased expression of glutamine metabolism proteins after GSK3203591 (n = 3).

Numerous genes involved in metabolic pathways such as glycolysis and glutamine metabolism were apparent amongst the shared DEGs and DSGs, a selection of which are highlighted in Fig. 6A. Notable DSGs included *PKM*, which has already been shown to undergo hnRNPA1-dependent alternative splicing in neuroblastoma [26] and *MLX*, encoding a MYCN transcriptional co-activator regulating glutamine metabolism genes in neuroblastoma [30]. Switching between *PKM1* and *PKM2* isoforms involves inclusion of exon 9 (*PKM1*) or exon 10 (*PKM2*) and is associated with the Warburg effect in cancer cells [31]. We confirmed AS of both *PKM* and *MLX*, together with the expected protein changes in all three MNA cell lines following GSK3203591 treatment (Fig. 6B–D). We also identified decreases in Mondo/MLX interacting protein (*MLXIP)*, previously shown to have interdependent expression with MLX [30], supporting the hypothesis that glucose and/or glutamine metabolism might be impaired following PRMT5 inhibition.

In order to test cellular utilization of glucose and glutamine following PRMT5 inhibition, we conducted stable isotope labelling using either uniformly labelled ^13^C_6_-glucose (U-[^13^C_6_]-Glc) or glutamine (U-[^13^C_5_]-Q). We observed decreases in glutamine-derived ^13^C incorporation in all three MNA neuroblastoma cell lines (Fig. 6E and Supplementary Figs. 5A and B), but no consistent changes in glucose incorporation into TCA intermediates following GSK3203591 treatment (Supplementary Figs. 6A–C). We therefore validated genes encoding glutamine metabolism proteins in our treatments, and found down-regulation of the glutamine transporter, *SLC1A5* and downstream glutamine metabolism genes *GOT1* and *CAD*. These genes have previously been linked with MYCN-associated glutamine addiction in neuroblastoma [32–34]. Transcript levels of *GLS*, encoding glutaminase, the main pathway ‘gatekeeper’ enzyme, were either unchanged or slightly increased, and *GLS2* levels were very low in all three lines (Fig. 6F). Immunoblotting confirmed that CAD and SLC1A5 protein levels were decreased after GSK3203591 treatment. Intriguingly, we found that GLS levels, specifically the GLS_KGA_ isoform, were decreased following PRMT5 inhibition (Fig. 6G). *GLS* encodes two isoforms of glutaminase, kidney type glutaminase (KGA, 65 kDa) and glutaminase C (GAC, 58 kDa). However, *GLS* was not included in our shared DEGs or DSGs, suggesting that PRMT5 may regulate GLS via post-transcriptional mechanisms.

Whilst our cell-line sensitivity assays demonstrated a clear MYCN-dependence (Fig. 1B), we were intrigued by the relative GSK3203591-sensitivity of the non-MNA line CHLA-15, especially as its post-therapy related line CHLA-20 has previously been shown to be GSK3203591-sensitive [14]. As these cell-lines are unusual amongst neuroblastoma lines in having *MYC* (c-MYC) amplification [35], and c-MYC-mediated global transcription is altered by glutamine deprivation [36], we posited that the relative sensitivity of CHLA-15/20 to GSK3203591 may be related to enhanced glutamine pathway dependence. We tested this possibility by treating a panel of neuroblastoma cell-lines with the GLS inhibitor CB-839 [37]. Strikingly, CHLA-15 cells exhibited dramatic apoptosis, in contrast to other neuroblastoma cell-lines (Supplementary Fig. 6D). Taken together, our metabolic studies strongly support the hypothesis that the PRMT5 dependency of MNA neuroblastoma is at least in part due to maintaining cancer cell fitness through glutamine addiction.

### 2.6 PRMT5 inhibition leads to alterations in the epitranscriptome

**Fig. 7:**
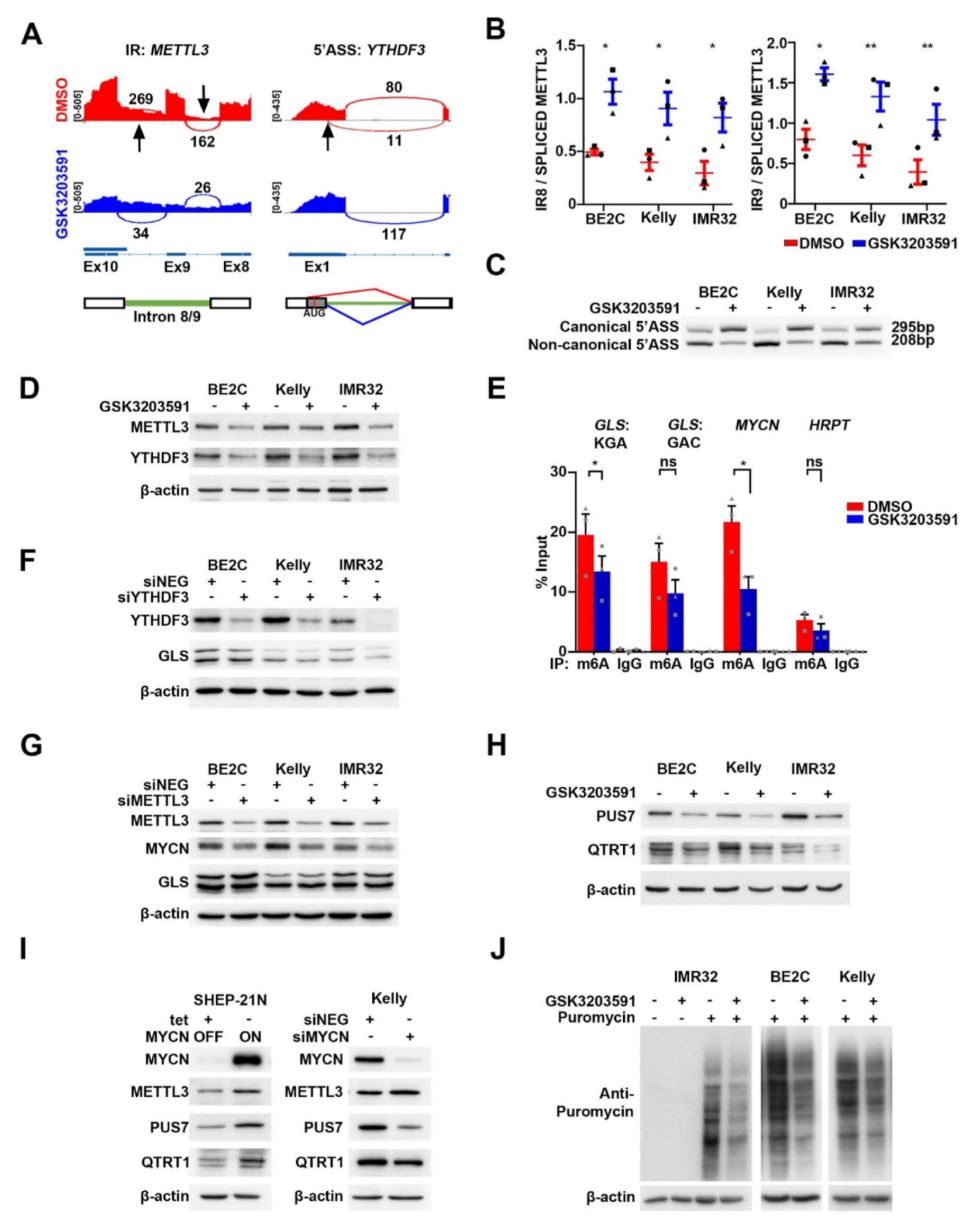
PRMT5 inhibition downregulates epitranscriptome regulators and decreases translation efficiency. **[A]** Representative sashimi plots of METTL3 intron retention of intron 8 and 9 (left) and YTHDF3 alternative 5′ splice site in exon 1 (right) after GSK3202591. **[B]** RT-qPCR showing increased intron retention of introns 8 and 9 in the *METTL3* transcript (n = 3, paired *t*-test, * = p < 0.05, ** = p < 0.005). **[C]** End point PCR validation of alternative 5′ splice site in exon 1 of YTHDF3 from the non-canonical splice site (DMSO) to the canonical splice site (GSK3203591) (n = 3). **[D]** Immunoblot showing decreased expression of METTL3 and YTHDF3 in BE2C, Kelly and IMR32 cells after GSK3203591 (n = 3). **[E]** Methylated RNA immunoprecipitation (MeRIP) performed using anti-m6A antibody demonstrates decreased m6A on *GLS* and *MYCN* transcripts detected by RT-qPCR in BE2C cells. *HRPT* was used as a control. (n = 3, mean ± SEM, paired *t*-test, * = p < 0.05). **[F]** Immunoblot showing decreased expression of GLS following YTHDF3 knockdown (n = 3). **[G]** Immunoblot showing decreased expression of MYCN following METTL3 knockdown (n = 3). **[H]** Immunoblot showing decreased expression of RNA modifier proteins PUS7 and QTRT1 after GSK3203591 (n = 3). **[I]** Immunoblot of cell extracts of SHEP-21N incubated with (MYCN off) or without (MYCN on) tetracycline (left) and Kelly cells transfected with siRNA targeting MYCN (right) probing for RNA modifier proteins PUS7, QTRT1 and METTL3 (n = 3). **[J]** SunSET assay showing decreased protein translation after GSK3203591 (n = 2).

The post-transcriptional regulation implied by our GLS analyses may occur via several mechanisms, including miRNA-mediated control or altered translational efficiency. Previous studies have shown c-myc regulates *AOPEP* which houses the GLS regulator miR-23 [38], however AOPEP was not in our DEGs. PRMT5 has already been shown to be involved in translational regulation via methylation of the hnRNPA1 protein, leading to enhanced hnRNPA1 binding to select internal ribosome entry sites (IRESs) and increasing translation [39]. Another possible mechanism suggested by our DSG geneset was diminution of METTL3 as a result of increased intron retention following GSK3203591 treatment (Fig. 7A). METTL3 is the key writer of m6A methylation on mRNA, which in turn lead to increased translation of mRNAs in cancer cells [40]. We also identified an alternative 5′ splice site in the m6A reader *YTHDF3* following GSK3203591 treatment (Fig. 7A). We confirmed intron retention of introns 8 and 9 of *METTL3* following PRMT5 inhibition (Fig. 7B) and validated *YTHDF3* mRNA 5′ splice site switching by end point PCR (Fig. 7C). Genome database analysis of *YTHDF3* suggests that the alternative 5’ splicing results in switching from an open reading frame commencing in exon 3 to the canonical YTHDF3 using the exon 1 AUG codon. Analysis of universal RNA, fetal kidney, fetal adrenal and neural crest cell lines confirmed the generality of the YTHDF3 splice-isoforms (Supplementary Fig. 7A). Alternative splicing events were accompanied by decreased METTL3 and YTHDF3 protein (Fig. 7D). We therefore assessed whether *GLS* mRNA was marked by m6A methylation, and whether this epitranscriptomic mark is altered by PRMT5 inhibition. As shown in Fig. 7E, m6A-methylated mRNA immunoprecipitation (MeRIP) confirmed both methylation of GLS mRNA and its reduction after GSK3203591 treatment of BE2C cells. GLS^KGA^ mRNA had higher m6A levels and a more significant decrease than GLS^GAC^, consistent with decreases in GLS^KGA^ protein observed in Fig. 6G. As GLS has been previously demonstrated to regulated by YTHDF1 [41] we knocked down YTHDF3 and discovered decreased GLS protein expression, reflecting our meRIP data KGA expression was decreased more than that of GAC (Fig. 7F, quantified in Supplementary Fig. 7B). These data demonstrate GLS expression is regulated by m6A modification and that the YTHDF3 reader is required for GLS translation.

Methylation of *MYCN* mRNA by METTL3 has been demonstrated recently [42], and we also observed a significant decrease of m6A methylation of *MYCN* mRNA following PRMT5 inhibition (Fig. 7E). We next sought to determine the effect of METTL3 knockdown on MYCN expression and identified decreased MYCN protein expression following knockdown (Fig. 7G). Developing on this evidence for post-transcriptional modifications, we further examined the intriguing link between PRMT5-MYCN and the epitranscriptome. Surveying of our DEGs and DSGs revealed potential decreases in several genes encoding epitranscriptomic modifiers, including the DEG *PUS7*, as well as the intron retention DSGs *QTRT1, NOP2* and *NSUN2* (Supplementary Figs. 7C–D). Interestingly *PUS7* is already identified as a MYCN-activated gene within the poor prognosis MYCN-157 signature [22]. Immunoblotting confirmed decreases of PUS7 (regulator of tRNA and mRNA pseudouridylation) and QTRT1 (catalyzes the trNA base-exchange of guanine with queuine) proteins following PRMT5 inhibition (Fig. 7H). Epitranscriptome modifier protein decreases following *MYCN* knockdown or increases accompanying *MYCN* induction in SHEP-21N cells were also confirmed for PUS7 and QTRT1 (Fig. 7I), further tightening the functional association of PRMT5-MYCN in post-transcriptional regulation.

As PRMT5 inhibition was demonstrated to have marked effects on epitranscriptomic regulators, we next explored whether global translation was affected by GSK3203591 in MNA neuroblastoma cells. For this we used the surface sensing of translation (SUnSET) assay which relies on incorporation of puromycin, a structural analogue of aminoacyl tRNAs, into nascent polypeptide chains, followed by probing cellular proteins with anti-puromycin antibodies. GSK3203591 consistently led to decreased puromycin incorporation, consistent with a global inhibition of translation and reduced cellular metabolism by PRMT5 inhibition (Fig. 7J).

Taken together, our *in vitro* data demonstrate that PRMT5 supports the fitness, survival, and proliferation of MNA neuroblastoma via diverse mechanisms including previously reported pathways such as mRNA splicing and DNA repair, but also novel routes such as MYCN-mediated transcriptional regulation, glutamine metabolism and epitranscriptome regulation.

### 2.7 Inhibition of PRMT5 in vivo significantly increases survival in a murine neuroblastoma model

**Fig. 8:**
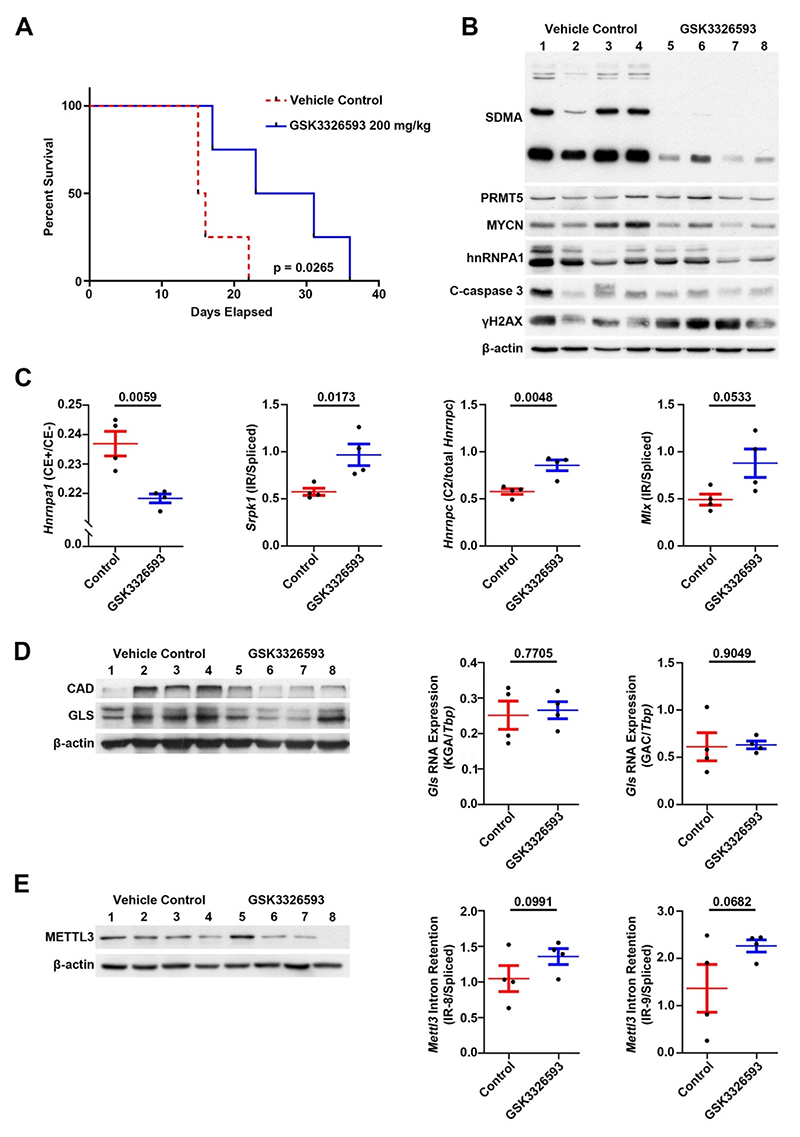
*In vivo* inhibition of PRMT5 in the *Th-MYCN* mouse neuroblastoma model significantly increases survival. **[A]** Kaplan-Meier showing survival of mice treated with GSK3326593 at 200 mg/kg (100 mg/kg twice a day) until maximum tumour burden was reached. Statistical significance for survival rates was determined using Log-rank (Mantel-Cox) test (control cohort n = 4; treated cohort n = 4). **[B]**. Immunoblot of protein extracts of mouse tumours showing inhibition of global SDMA in GSK3326593 treated tumours confirming on-target specificity of GSK3326593. **[C]** RT-qPCR of a selection of alternative splicing events first identified *in vitro*, using RNA extracted from Th*-MYCN* mouse tumours after PRMT5 inhibition (control cohort n = 4; treated cohort n = 4, unpaired *t*-test, exact p-value displayed). **[D]** Immunoblot of protein extracts of mouse tumours from control and GSK3326593 treated mice showing decreased GLS and CAD after GSK3326593 treatment (left). RT-qPCR (right) shows no significant change of *Gls* isoforms KGA/GAC mRNA (control cohort n = 4, treated cohort n = 4, unpaired *t*-test, exact p-value displayed). **[E]** Immunoblot (left) of protein extracts from mouse tumours showing decreased METTL3 protein after GSK3326593 treatment. RT-qPCR (right) demonstrating increased intron retention of intron 8 and 9 in the *Mettl3* transcript (control cohort n = 4, treated cohort n = 4, unpaired *t*-test, exact p-value displayed).

Following our *in vitro* analyses, we assessed the *in vivo* efficacy of PRMT5 inhibition. For this we used the *Th-MYCN* mouse model for neuroblastoma, and an analogue of GSK3203591, namely GSK3326593, which has better characteristics for *in vivo* work. The *Th-MYCN* GEMM was generated by targeting MYCN expression to the neural crest under the regulation of the tyrosine hydroxylase promoter. Importantly, these tumours faithfully recapitulate the pathologic and molecular features of the human disease and have been used extensively to evaluate novel therapeutic strategies aimed at treating the poor-outcome group of neuroblastoma patients [43]. A significant improvement in survival rates was apparent in mice dosed with 100 mg/kg GSK3326593 twice a day (Log-rank (Mantel-Cox) test, p = 0.0265) (Fig. 8A). Parallel treatments for tumour response studies were harvested following five days of GSK3326593 or vehicle treatment and used for molecular analyses. Immunoblotting with anti-SDMA antibodies showed clear decreases in SDMA, confirming the on-target effect of GSK3326593, together with a small but consistent decrease of Mycn protein. Apoptosis markers were not increased following Prmt5 inhibition, but γH2AX increased, indicating elevated DNA damage (Fig. 8B). We confirmed several of our DSGs in mouse tumours, including *Hnrnpa1, Srpk1, Hnrnpc*, and *Mlx* (Fig. 8C). We further demonstrated decreases of glutamine pathway enzymes Gls and Cad proteins, although *Gls* mRNA was unaltered (Fig. 8D). We therefore assessed Mettl3 protein and *Mettl3* intron retention and observed trends of decreased Mettl3 protein and increased *Mettl3* intron retention following GSK3326593 treatment (Fig. 8E), consistent with our findings in MNA cell-lines, where GLS expression is subject to epitranscriptomic post-transcriptional control.

In summary, our *in vivo* data demonstrate that GSK3326593 is a highly selective Prmt5 inhibitor, and that inhibition of Prmt5 in *MYCN*-dependent mouse neuroblastoma compromises the fitness and proliferation of tumour cells, resulting in their increased survival.

## 3 Discussion

The importance of PRMTs in tumourigenesis is reflected in the increasing interest in developing targeted therapeutics for this family of enzymes, with several studies demonstrating that PRMTs are a synthetic or collateral vulnerability in cancer [44]. The association of PRMTs with key oncogenes is best demonstrated in *Eμ-myc* driven lymphomagenesis in transgenic mice where c-myc requires Prmt5-mediated regulation of the spliceosome [17], and PRMT5 also acts with several other oncogenic pathways [45]. In the case of solid tumours, our group previously demonstrated that neuroblastoma cells over-expressing the *MYCN* oncogene are highly susceptible to loss of PRMT5 [5], prompting this preclinical assessment of the selective PRMT5 inhibitors GSK3203591 and GSK3326593 in neuroblastoma. We show that MNA neuroblastoma is sensitized to PRMT5 inhibition *in vitro* and *in vivo*, adding to the recent evidence for the spliceosomal vulnerability of neuroblastoma [27,28,46, 47]. Our study consolidates and extends the links previously made between MYCN activity in neuroblastoma and spliceosomal vulnerability [26,28,47]. Crucially, our mechanistic analyses identify and characterize epitranscriptomics and glutamine metabolism as actionable downstream pathways downstream of PRMT5-regulated altered alternative splicing programs.

Analysis of our neuroblastoma cell line panel demonstrated increased sensitivity of MNA neuroblastoma lines to GSK3203591, irrespective of their *TP53* mutational status. For example, CHP-212, IMR32 and NGP cell lines have wild-type *TP53*, whereas others such as LAN-1, Kelly and BE2C have mutant *TP53*. Activation of the p53 pathway was previously shown to increase sensitivity to GSK3203591 due to alternative splicing of *MDM4* transcripts induced by PRMT5 inhibition, leading to skipping of exon 6 which encodes the p53 interaction domain [19]. Whilst we observed exon skipping of exon 6 of *MDM4* mRNA in neuroblastoma cell lines too, our GSK3203591 sensitivity spectrum suggests that p53 activation is not the major determinant of drug sensitivity in MNA neuroblastoma. Whilst PRMT5 levels were unchanged following GSK3203591 treatments, we observed decreases of MYCN and E2F1. PRMT5 was previously shown to methylate and stabilize E2F1 [10] so the observed decrease is likely attributable to altered MYCN regulation of *E2F1* which is an established MYC/MYCN target gene [23–25]. Significantly more apoptosis was seen in MNA cell-lines, although overall cell death was modest even in sensitive cell-lines. The MYCN-dependency for GSK3203591 sensitivity is further strengthened by the SHEP-21N isogenic cell-line model with tetracycline-regulated MYCN where high MYCN led to decreased clonogenic survival, increased apoptosis and elevated PRMT5 and SDMA. PRMT5 has previously been shown to be a c-myc [17] and MYCN target [21], consistent with our protein level evidence (Fig. 2F–G and (5)). Together, our genetic interference [5] and pharmacological inhibition data strongly suggest that PRMT5 is synthetically lethal with MYCN in neuroblastoma. This spliceosomal vulnerability likely extends to other components of the spliceosomal machinery such as HNRNPA1 [26], RBM39 [28] and SNRPD3 [47], as well as other cancers such as Wilms’ tumour, where our transcriptomic analyses revealed MYCN regulation of spliceosome regulators including *SNRPD1* and *WDR77* [48].

Apart from *TP53* status (discussed above), the most compelling PRMT5 collateral vulnerability reported to date is with loss of the *MTAP* (methylthioadenosine phosphorylase) gene leading to elevation of cellular methylthioadenosine, resulting in PRMT5 inhibition [49,50]. However, *MTAP* lesions have not been shown in neuroblastoma – in fact *MTAP* has been reported to be transactivated by MYCN [22]. It will therefore be important to evaluate the dependency of other MYCN-driven malignancies on PRMT5 to establish the generality of the MYCN-PRMT5 axis vulnerability.

Inhibition of PRMT5 led to a dramatic curtailment of *MYCN*-activated genes, including gene signatures strongly associated with poor prognosis in neuroblastoma [22,51], BRD4 inhibition of neuroblastoma with JQ-1 [52] and cell-cycle resolved MYCN-activated genes from RNA sequencing in cell-cycle-synchronized neuroblastoma cells [53]. Whilst the number of genes whose transcripts increased after GSK3203591 treatment was similar to decreased transcripts, meta analysis with gene signatures strongly suggested that the major determinants for GSK3203591-mediated growth inhibition were the genes decreasing after PRMT5 inhibition. PRMT5 is known to exert epigenetic silencing through histone 3 arginine methylation [7], but our DEGs suggest that epigenetic derepression via PRMT5 inhibition is not a major route of drug action in MNA neuroblastoma. Global decreases of the PRMT5 histone modifications were also previously shown to be unaffected by GSK3203591, although specific loci may be epigenetically regulated [18]. GSK3203591 treatment led to slight decreases of MYCN protein *in vitro* and *in vivo*, but not dramatically as we previously observed after PRMT5 knockdowns [5]. This suggests that the PRMT5-MYCN protein interaction may stabilize MYCN independent of PRMT5 catalytic activity similar to the manner in which Aurora kinase A [54] and EZH2 [55] have been shown to stabilize MYCN protein. Activity of the MYC-family transcription factors can be influenced by many interactions including MAX, MXD1-4 and MNT [56] but these were not altered amongst our DEGs following GSK3203591 treatment. However, we did observe splicing related effects on another MYCN transcriptional co-factor MLX (discussed below) which may explain, at least in part, the profound effect of PRMT5 inhibition on MYCN transcriptional programmes. As well as the decreased fidelity of splicing following PRMT5 inhibition demonstrated by our data, PRMT5 may also positively regulate transcription through interactions with SWI/SNF chromatin remodellers [57]. In this regard, it is interesting to note that MYCN requirement of remodelling proteins such as SMARCE1 has recently been demonstrated [58].

PRMT5 is known to be crucial in the assembly of spliceosomal complexes that regulate alternative splicing. Key substrates for PRMT5 include the Sm proteins, and restriction of their symmetrical dimethylation results in incorrect assembly into small nuclear ribonucleoprotein particles (snRNPs). This results in the failure of complexes to recognize weak 5′-splice sites, inducing a variety of splicing defects, in particular intron retention [16,17]. Consistent with this, GSK3203591 treatment resulted in numerous alternative splicing events shared between our three MNA cell-lines, approximately half of which were intron retentions. Similar predominance of intron retention following pharmaceutical inhibition of PRMT5 has been shown in glioblastoma and haematopoietic cells [59,60]. Whilst a significant overlap was observed between alternative splicing events resulting from PRMT5 inhibition and RBM39 loss, there were still many differences which may be attributable to the diversity of functions performed by PRMT5 (discussed above) and RBM39, such as transcriptional cofactor roles [61].

There was a very high correlation between our DEGs and DSGs and the MYCN-157 signature. This signature was reported as a stronger prognostic indicator than *MYCN* amplification alone, and our data shows correlations where *MYCN* transcript levels were relatively low but MYCN protein levels were significant [22]. Whilst high c-MYC levels have also been shown to correlate with high-risk neuroblastoma [4], positive *MYC* correlation with our DEGs or DSGs was not observed. However, our data with the CHLA-15 line, together with reports such as the sensitivity of c-MYC over-expressing pancreatic cancers to PRMT5 inhibition [62] suggest that threshold and context-dependent effects are important in c-MYC-driven cancers.

The JUM bioinformatic pipeline yielded strongly validated alternative splicing hits encompassing many key regulatory pathways, including DNA repair and mRNA processing, as shown in glioblastoma [59]. The multifunctional protein hnRNPA1, already known to be post-translationally modified by PRMT5, CARM1 and PRMT7 [39,63], was consistently shown to switch from an alternatively spliced (+ exon 8) isoform to a minus exon 8 form at RNA and protein level following GSK3203591 treatment. Whilst little is known about this isoform, it has been demonstrated to have much stronger binding to RNA [64] and is also expressed at elevated levels in chronic myelogenous leukemia compared to control cells [65]. Given that hnRNPA1 is involved in many essential pathways including transcriptional and translational regulation, splicing and telomere maintenance [66], it will be of interest to examine the tumorigenic role of hnRNPA1(+ exon 8) in future. Alternative splicing of several other HNRNP genes was also apparent, suggested that PRMT5 regulates the intricately regulated assembly of hnRNP complexes that regulate splicing [67].

PRMT5 regulates DNA repair through multiple mechanisms such as direct methylation of RUVBL1 [68] and alternative splicing of *KAT5* [69]. Our study reveals several other genes encoding DNA repair pathway proteins undergo splicing alterations that accompany enhanced DNA damage by GSK3203591, including *TIMELESS* [70] and *DONSON* [71]. PRMT5 inhibition has recently been shown to enhance the sensitivity of breast and ovarian cancers to PARP inhibitors [72], and the convergence of alterations in DNA replication and repair genes observed in our data suggests that similar combinations could be effective against poor prognosis neuroblastoma. MYCN-driven replicative stress has already been shown to increase susceptibility to PARP inhibition in neuroblastoma [73], further rationalizing the combination of PRMT5 and PARP inhibition for MNA neuroblastoma.

Having shown that PRMT5 inhibition triggers widespread splicing changes, possibly through master nodes such as hnRNP proteins, we endeavoured to characterize key downstream events. As we mostly observed inhibition of cell growth rather than pronounced apoptosis, suggesting that PRMT5 may support the metabolic fitness of MNA neuroblastoma, we were intrigued by the consistent PKM2-PKM1 splicing switch observed after PRMT5 inhibition. PKM2 drives aerobic glycolysis in preference to oxidative phosphorylation [31] and the isoform switch has been shown to depend on hnRNPA1 [26,74]. Despite strong mRNA and protein validation of the PKM isoform switching, metabolic tracing did not show any consistent effects on glucose metabolism; additionally oxidative phosphorylation was also unaltered (data not shown). However, PKM2 also phosphorylates non-metabolic substrates, for example stabilizing Bcl-2 [75] and influencing transcription via phosphorylation of histone 3, already demonstrated in neuroblastoma [76], and perturbation of these pathways may contribute to GSK3203591-mediated growth inhibition. Numerous other DEGs and DSGs identified by our transcriptomic analyses encode metabolic pathway proteins. Given the MNA neuroblastoma-specific inhibition spectrum exhibited by GSK3203591 and the addiction of MNA neuroblastoma to glutamine [32,77], the convergence of DEGs and DSGs on genes in this pathway strongly supports the premise that PRMT5 contributes to the metabolic fitness of MNA neuroblastoma via augmenting glutamine metabolism. Specifically, PRMT5 inhibition can impede glutamine metabolism via (i) the coupling of glutamine sensing and transcription via intron retention of *MLX*, (ii) negatively influencing the MYCN-mediated transcription of glutamine pathway genes (*CAD, SLC1A5, GOT1*) and (iii) epitranscriptomic regulation of GLS following alternative splicing of regulators METTL3 and YTHDF3. Impaired glutamine metabolism may also affect the MYCN-regulated transcriptome through decreasing nucleotide synthesis downstream of glutamine anaplerosis [78]. Intriguingly, RBM39 function was linked with regulating glucose metabolism [28], and recently RBM39 was also shown to modulate metabolism via arginine-sensing [79]. Together with our study, this suggests an important generality of splicing factors regulating metabolic pathways.

Whilst many PRMT5 inhibitor induced AS events were also observed in non-MNA cell lines, drug susceptibility was not apparent, attributable at least in part to non-MNA neuroblastoma not having a dependency on glutamine for survival and proliferation, with glutamine deprivation triggering apoptosis only in MNA neuroblastoma [34]. Interestingly, we found that the non-MNA neuroblastoma cell-line CHLA-15 with *MYC* amplification was unusually sensitive to GLS inhibition with CB-839, in contrast to other lines. This may be due to effects on global transcription via RNA polymerase II stalling as a result of glutamine-dependant changes in nucleotide levels leading to R-loop formation and DNA damage. These effects are dependant on glutamine-sensing by the c-MYC 3′-UTR [36]. As CHLA-15 have functional p53, this may trigger the marked apoptosis we observed. In contrast the MNA neuroblastoma lines likely require several glutamine metabolism hits, as characterised in our study. Whilst MYC amplification is very rare in neuroblastoma [80], our observations with CHLA-15 cells may have clinical relevance for some patients.

MYCN-dependent transcription of genes such as *SLC1A5, CAD* and *GOT1* has been previously reported, but regulation of the main pathway ‘gatekeeper’ *GLS* has not. Although c-MYC has been shown to regulate GLS expression via miR-23 [38], we note that miR-23 levels are not significantly different in a comparison of MNA and non-MNA neuroblastomas [81]. Whilst we do not exclude this possibility, our studies demonstrate that GLS levels are indeed regulated by MYCN-PRMT5 through alternative splicing and epitranscriptomic pathways via the m6A reader YTHDF3. PRMT5 has recently been shown to control intron retention of METTL3 in blastic plasmacytoid dendritic cell neoplasm [82], similar to our observations in neuroblastoma. Interestingly, METTL3 knockdown alone did not cause GLS protein to decrease, possibly because METTL3 loss may cause increased 3′-UTR lengthening of GLS [83] thus favouring KGA isoform expression. The combination of decreased *GLS* mRNA m6A-methylation and decreased YTHDF3 protein expression support translational inhibition of *GLS* mRNA by GSK3203591 contributing to altered glutamine metabolism. Whilst the read-outs of epitranscriptome marks such as m6A-methylation are very diverse [84], epitranscriptomic and mRNA translation control of GLS is supported by YTHDF1-dependent GLS regulation in colon cancer [41]. Notably, our work suggests that PRMT5 is at the crux of epitranscriptome regulation as we also observed numerous other RNA writers and readers as targets for PRMT5-dependent splicing, including *QTRT1, NOP2* and *NSUN2*. The multiple levels of RNA regulation via these proteins, and of the hnRNPs, also known to interact with the epitranscriptomic machinery [85], suggests that the PRMT5-MYCN axis is also integral in proteostasis in neuroblastoma, which is supported by our global translation analyses. Intriguingly, MYCN-regulated translation has recently been reported as a therapeutic vulnerability in medullo-blastoma [86], and our studies support this possibility in high-risk neuroblastoma. Importantly, highly selective epitranscriptic inhibitors are now emerging [87], and the interdependence of splicing and epitranscriptomic pathways suggests that drug combinations selectively targeting both pathways may be highly efficacious. Our work also highlights the interplay of arginine methyltransferases and RNA methylation, as previously suggested for PRMT1 and METTL14 [88].

PRMT5 inhibition using GSK3326593 led to a significant improvement in survival in the *Th-MYCN* neuroblastoma mouse model and excellent target engagement was observed with strong SDMA reduction. Pathways identified by our *in vitro* analysis as being downstream of the MYCN-PRMT5 axis, including splicing, epitranscriptomics, DNA damage and glutamine metabolism, were also affected *in vivo*. Cell death and tumour regression were not apparent, in contrast to the apoptosis observed *in vitro*. The extended survival we observed may be attributable to several factors including (i) the metabolic interactions with the tumour microenvironment, demonstrated in a model of ovarian cancer, where inhibition of stromal glutamine synthetase as well as tumour glutaminase were required to reduce tumour burden [89], (ii) curtailment of metastasis, as demonstrated in neuroblastoma xenografts following PRMT5 inhibition [14] and (iii) immune responses arising from neoantigens following mRNA splicing [90].

Clinically, GSK3326595, the sister compound to those we have tested, has been assessed in the METEOR-1 clinical trial (NCT02783300) and has shown benefit in some adult cancers such as adenoid cystic carcinoma, with tolerable adverse effects such as fatigue, anaemia, and nausea [91]. No paediatric patients were enrolled. Although the efficacy of monotherapy was restricted, neuroblastoma may be more responsive than other tumours including diffuse midline glioma where no survival benefit was observed [92]. Notably, a comparison of neuroblastoma with 25 other cancers using DEMETER (https://depmap.org/rnai/) showed that neuroblastoma is the cancer most susceptible to depletion of spliceosomal proteins [27]. Additionally, our study outlining the pleiotropic effects of PRMT5 inhibition induced transcriptome changes on cell fitness, together with inhibition of metastasis via Akt signalling [14], indicate that PRMT5 inhibition will be especially useful as part of combination therapies. Such studies are already yielding promising results in other cancers [93,94] and the detailed mechanistic analyses presented here rationalize new actionable pathways for combination therapies of cancers with spliceosomal vulnerabilities.

## Materials and methods

### 4.1 Cell culture

Sources of neuroblastoma and immortalised disease-free cell lines used in this study are detailed in Supplementary File 2 – Cell lines. LAN-1, LAN-5, Kelly, and GIMEN were cultured in RPMI 1640 (Gibco), and CHLA-15, CHP-212, IMR32, NGP, SK-N-BE(2)C (BE2C), SHEP, SH-SY-5Y, SK-N-AS, SH-IN, NBL-S, LAN-6, RPE-1, NF-TERT, and NF-730 were cultured in Dulbecco’s modified eagle’s medium (DMEM):F12-HAM (Sigma). Both media were supplemented with 10 % (v/v) FBS (Life technologies), 2 mM L-glutamine, 100 U/mL penicillin, 0.1 mg/mL streptomycin, and 1 % (v/v) non-essential amino acids. SH-EP-Tet21N (SHEP-21N) were cultured in RPMI 1640 (Gibco), supplemented with 10 % (v/v) tetracycline-free FBS (Life technologies), 2 mM L-Glutamine, 100 U/mL penicillin, 0.1 mg/mL streptomycin, and 1 μg/mL tetracycline. Cell counts were measured using a Countess automated cell counter (Thermo Fisher Scientific). All cell lines were routinely tested for mycoplasma contamination and were confirmed to be mycoplasma negative. Main genotype details of cell lines are given in Supplementary File 2 – Cell lines. Human biological samples were sourced ethically, and their research use was in accord with the terms of the informed consents under an IRB/REC approved protocol.

### 4.2 Drug treatments, short-interfering RNA knockdowns, and cell proliferation

Cells were seeded and allowed to adhere prior to treatment with GSK3203591 (Selleckchem, cat. no. S8111). Control cells were treated with equivalent DMSO concentration. Scaled-up preparative GSK3203591 treatments were generally 1 μM–3 μM for 96 h unless otherwise indicated. Transient siRNA knockdowns were performed by using short interfering RNA (Supplementary File 2 - siRNA). Reverse transfections were performed using 25 nM siRNA and Lipofectamine RNAiMAX (Invitrogen) complexed in OptiMEM media (Invitrogen) and added to cell suspensions prior to seeding and incubated for at least 48-h before cell lysis. For knockdown and drug combination experiments, cells were reverse-transfected, drug treated after 24 h, then harvested after 96 h. Cell proliferation was measured using the IncuCyte ZOOM live-cell analysis system with images taken every 4-h. The glutaminase-specific inhibitor CB-839 was purchased from APExBIO (cat. no B4799).

### 4.3 MTT cell viability and colony formation assays

GSK3203591 (Selleckchem) survival assays were carried out over six days. Cells were seeded in 96-well plates at a cell density ranging from 500 to 3000 cells/well and treated the next day in triplicate with a serial dilution of GSK3203591. Thereafter, 10 μL of MTT (5 mg/mL) (Sigma) was added/well, followed by 40 μL of SDS lysis buffer (10 % SDS (w/v), 1/2500 (v/v) 37 % HCl) after 3 h had elapsed. Following overnight incubation at 37 °C, the plates were read at 570 nm and 650 nm, using SpectraMax 190 plate reader (Molecular Devices). For colony formation assays, cells were seeded into 6-well plates (1 x 10^3^ cells/well) and allowed to adhere for 24-h before being treated with GSK3203591 or DMSO equivalent. Cells were re-dosed with GSK3203591 or DMSO every 96h. After 17-days cells were fixed in 4 % paraformaldehyde and stained with methylene blue.

### 4.4 Protein extraction and immunoblotting

Floating cells were collected and attached cells trypsinised and then combined and centrifuged at 4 °C collected before being lysed in Radioimmunoprecipitation assay (RIPA) buffer with added protease and phosphatase inhibitors and sonicated on high for 30 s off 30 s on for 3 min (Diagenode Bioruptor). The protein concentration was determined using the Micro BCA TM protein assay kit (Thermo Fisher). Immunoblotting was performed as described previously [5] using antibodies in Supplementary File 2 - antibodies.

### 4.5 RNA extraction, reverse transcription, PCR assays

RNA was extracted from attached cells using QIAzol and the miR-Neasy kit (QIAGEN) according to the manufacturer’s instructions, including DNAse treatment. cDNA synthesis was performed using Superscript IV cDNA synthesis kit (Invitrogen). Quantitative PCR (5 ng cDNA/well) was performed by using QuantiNova kit on Mx3500P PCR machine (Stratagene). Gene-specific primers were used for end-point PCR (HotStarTaq Plus DNA Polymerase; Qiagen), to detect inclusion or exclusion of alternative exons, after electrophoresis on agarose gels (2 %). The oligonucleotide primers used to detect target gene expression and alternative splicing events are listed in Supplementary File 2 – primers.

### 4.6 Cell cycle analysis

Cells were seeded and treated with GSK3203591 or DMSO equivalent for 24–96 h. Cells were trypsinised, washed in PBS and added dropwise to ice cold 70 % ethanol and kept at − 20 °C for at least 2-h. For propidium-iodide only labelling 15 min 37 °C RNase digestion (final 0.5 μg/mL) and 20 μg/mL propidium iodide staining were performed and flow cytometry analysis was performed with 10^4^ cells/sample as previously described [5], and data analysed using FlowJo software v10. For BrdU incorporation analysis, 10 μM BrdU (Sigma) was added to the cells and incubated for 30 min. The cells were then trypsinised, centrifuged, washed in ice-cold PBS, and fixed in 70 % ice-cold ethanol at − 20 °C for at least 24 h. On the day of flow cytometric analysis, the cells were centrifuged, the ethanol was aspirated, and the cells were resuspended in 2N HCl containing 0.1 % Triton X-100. Cells were incubated at room temperature for 30 min. After centrifugation, the HCl was neutralized with 0.1M Na2B4O7 (pH 8.0), followed by centrifugation and resuspension in 0.1 % BSA. The cells were then centrifuged again and resuspended in a BrdU antibody solution (BD-347580) prepared by diluting 20 μl of antibody in 1 ml of PBS containing 0.1 % BSA and 0.05 % Tween-20 (1:50 dilution). The cells were incubated with the BrdU antibody for 1 h. After washing with PBS, Alexa Fluor-conjugated goat anti-mouse secondary antibody (ThermoFisher, A11029, 1:500) was added and incubated in the dark at room temperature for 30 min. Finally, 15 min 37 °C RNase digestion (final 0.5 μg/mL) and 20 μg/mL propidium iodide staining were performed Samples were analysed using a BD LSRFortessa, and data analysis was performed using FlowJo software v10.

### 4.7 RNA sequencing

RNA was extracted as above and quantified using Nanodrop (ND1000) and quality confirmed using an Agilent ScreenTape RNA assay. Two biological replicates were used for RNA-seq of each condition with the paired-end option of 100bp reads on BGIseq-500 (BGI, Shenzhen, China). Differential gene expression bioinformatics and alignments were as previously described [48]. Alternative splicing analysis was conducted with The Junction Usage Model (JUM) splicing analysis package [95]. RNA-seq reads were aligned to the human genome (hg38) with the Spliced Transcripts Alignment to a Reference (STAR) protocol [96]. We used a p=<0.05 and a ΔPSI >0.05 cut-off for assignment of differentially spliced genes (DSGs). Gene expression analyses of published neuroblastoma datasets and Kaplan-Meier analyses were performed by using the R2 Genomics Analysis and Visualization Platform (http://r2.amc.nl).

### 4.8 Irradiation and confocal microscopy

Cells were seeded on rat tail collagen (10 μg/mL) coated coverslips 24-h prior to treatment with GSK3203591/DMSO for 48 h. Cells were then exposed to 2.5 Gy irradiation and incubated for 1- or 24-h in media containing GSK3203591. Cells were fixed using 4 % paraformaldehyde and washed 3x with PBS. Coverslips were permeabilised for 25 min in PBS containing 0.1 % Triton-X100 (Merck, UK), blocked in PBS containing 10 % (v/v) BSA + 0.1 % (v/v) Triton-X100 (both Merck, UK) and primary antibodies (γH2AX, Cell Signalling Technologies, 1:200) diluted in antibody buffer (PBS containing 2 % (v/v) BSA + 0.1 % (v/v) Triton-X) were applied at 4 °C overnight. Coverslips were washed 3x PBS and secondary antibodies (Alexa Fluor™ 555 anti-rabbit, A-21428, 1:1000) applied in antibody buffer for 1-h in the dark at room temperature, washed with PBS and incubated 5 min with PBS containing 2 μg/mL Hoechst (33342, Fisher Scientific, UK) and washed in PBS and briefly in ddH2O then mounted on glass slides using ProLong GoldTM anti-fade glass mounting medium (P36980, Fisher Scientific, UK). Z-stack images were acquired using a Leica SP8 confocal microscope at magnifications indicated in legends using 2048 × 2048 pixel resolution. Foci per nucleus were quantified using CellProfiler 4.2.6 using maximal orthogonal projection of Z-stacks.

### 4.9 m6A-methylated RNA immunoprecipitation (MeRIP)-qPCR

Cells were treated with GSK3203591/DMSO equivalent for 96 h prior to being trypsinised and collected. Total RNA was prepared using RNeasy Midi kit (Cat# 75144, QIAgen, UK) as per manufacturer’s instructions and DNase digest was performed on column using RNase free DNase set (Cat# 79254, QIAgen, UK). RNA was quantified using NanoDrop1000 and 75 μg total RNA was used to purify mRNA using Dynabeads® mRNA Purification Kit (Cat# 61006, Fisher, UK) and RNA clean up performed using RNeasy MinElute Cleanup Kit (Cat# 74204) both as per manufacturer’s instructions. N^6^-Methyladenosine RNA immunoprecipitation (meRIP) was performed using Magna MeRIP™ m6A Kit (Cat# 17–10499, Sigma-Aldrich, UK) as per manufacturer’s instructions. Briefly, protein A/G beads were washed in 1x IP buffer three times and coupled to 5 μg m6A antibody (MABE1006) or IgG (CS100621) at room temperature for 30-min. Beads were then washed three times and resuspended in meRIP reaction mixtures containing 0.5 μg unfragmented in-tact mRNA and 1 % (v/v) RNase inhibitor in 1x IP-buffer and incubated with rotation for 2 h at 4 °C. Beads were washed three times in IP buffer and transferred to a clean tube. Immunoprecipitated transcripts were eluted by resuspending beads in elution buffer containing 6.67 mM N^6^-Methyladenosine, 5′-monophosphate sodium salt (CS220007) and RNase inhibitor in 1x IP buffer and incubated for 1 h with shaking at 4 °C. Eluates were transferred to clean tubes and RNA clean up performed using RNeasy MinElute Cleanup Kit (Cat# 74204) as per manufacturer’s instructions. cDNA was synthesised as above on 10 % (50 ng) mRNA input, m6A-IP and IgG-IP samples and then analysed using RT-qPCR, as above.

### 4.10 Stable isotope labelling

Cells were seeded 24-h prior to treatment with 2.5 μM GSK3203591 for 72-h before being washed with PBS and incubated for 8-h with DMEM media supplemented with 10 % dialysed FBS, 1 % pen/strep, 10 mM glucose (unlabelled or ^13^C_6_-glucose labelled), 2 mM glutamine (unlabelled or ^13^C_5_-glutamine labelled). Media was removed and cells washed twice in ice-cold saline. Plates were then placed on dry ice and scraped into 800 μL ice-cold 80 % LC-MS grade methanol, centrifuged at 15,000x*g* for 10 min 4 °C and stored at −80°C.

Cellular metabolites were extracted and analysed by gas chromatography-mass spectrometry (GC-MS) using protocols described previously [97]. Metabolite extracts were derived using N-(tert-butyldimethylsilyl)-N-methyltrifluoroacetamide (MTBSTFA) as described previously [98]. An internal standard, D-myristic acid (750ng/sample), was added to metabolite extracts. Mass isotopomer distribution was determined using a custom algorithm developed at McGill University [97].

### 4.11 SunSet assay

Translation of nascent protein was measured using the previously described SUrface SEnsing of Translation (SUnSET) assay [99]. Cells were seeded 24-h prior to treatment with GSK3203591/DMSO for 72h then pulsed with 1.25 μM puromycin for 1-h prior to cell lysis and protein extraction (as above). Protein extracts of puromycin pulsed and non-puromycin pulsed control cells were subject to immunoblot (as above) using anti-puromycin antibody (clone 12D10, 1:1000, Sigma).

### 4.12 In vivo evaluation of GSK3326593 in Th-MYCN GEMM mice

GSK3326593 which has similar potency to GSK3203591 (personal communication, GlaxoSmithKline) was obtained under MTA from GlaxoSmithKline. The study was performed using both male and female hemizygous mice, which developed palpable tumours at 50–130 days with a 25 % penetrance. Transgenic Th-*MYCN* mice were genotyped to detect the presence of human *MYCN* transgene. Tumour development was monitored weekly by palpation by an experienced animal technician. Mice with palpable tumours at >3 mm were treated with either GSK3326593 at 100 mg/kg, twice per day by oral gavage or vehicle (0.5 % methylcellulose) twice per day by oral gavage. Mice were housed in specific pathogen-free rooms in autoclaved, aseptic microisolator cages (maximum of four mice per cage). Mice were allowed access to sterile food and water ad libitum. All experiments were approved by The Institute of Cancer Research Animal Welfare and Ethical Review Body and performed in accordance with the UK Home Office Animals (Scientific Procedures) Act 1986, the United Kingdom National Cancer Research Institute guidelines for the welfare of animals in cancer research and the ARRIVE (animal research: reporting *in vivo* experiments) guidelines.

### 4.13 Statistical tests

The normality of datasets ≥3 datapoints was determined using a Shapiro-Wilks test. Normally distributed datasets were analysed using a *t*-test with a significant p-value of 0.05 and data were plotted using GraphPad Prism 9.4.0. Statistical tests, sample size and the number of independent biological replicates performed for specific experiments are detailed in figure legends.

## Supplementary Material

Supplementary data to this article can be found online at https://doi.org/10.1016/j.canlet.2024.217263.

Supplementary data

## Data Availability

RNA sequencing data is available from the European Nucleotide Archive (ENA) accession PRJEB72851/ERP157650.
