# Supplementary material for: Spliceosomal vulnerability of MYCN-amplified neuroblastoma is contingent on PRMT5-mediated regulation of epitranscriptomic and metabolomic pathways
Source: Cancer Lett. Author manuscript; Available in PMC 2025 Aug 27. (PMC7618037; doi:10.1016/j.canlet.2024.217263)

**SUPPLEMENTARY FIGURE 1**


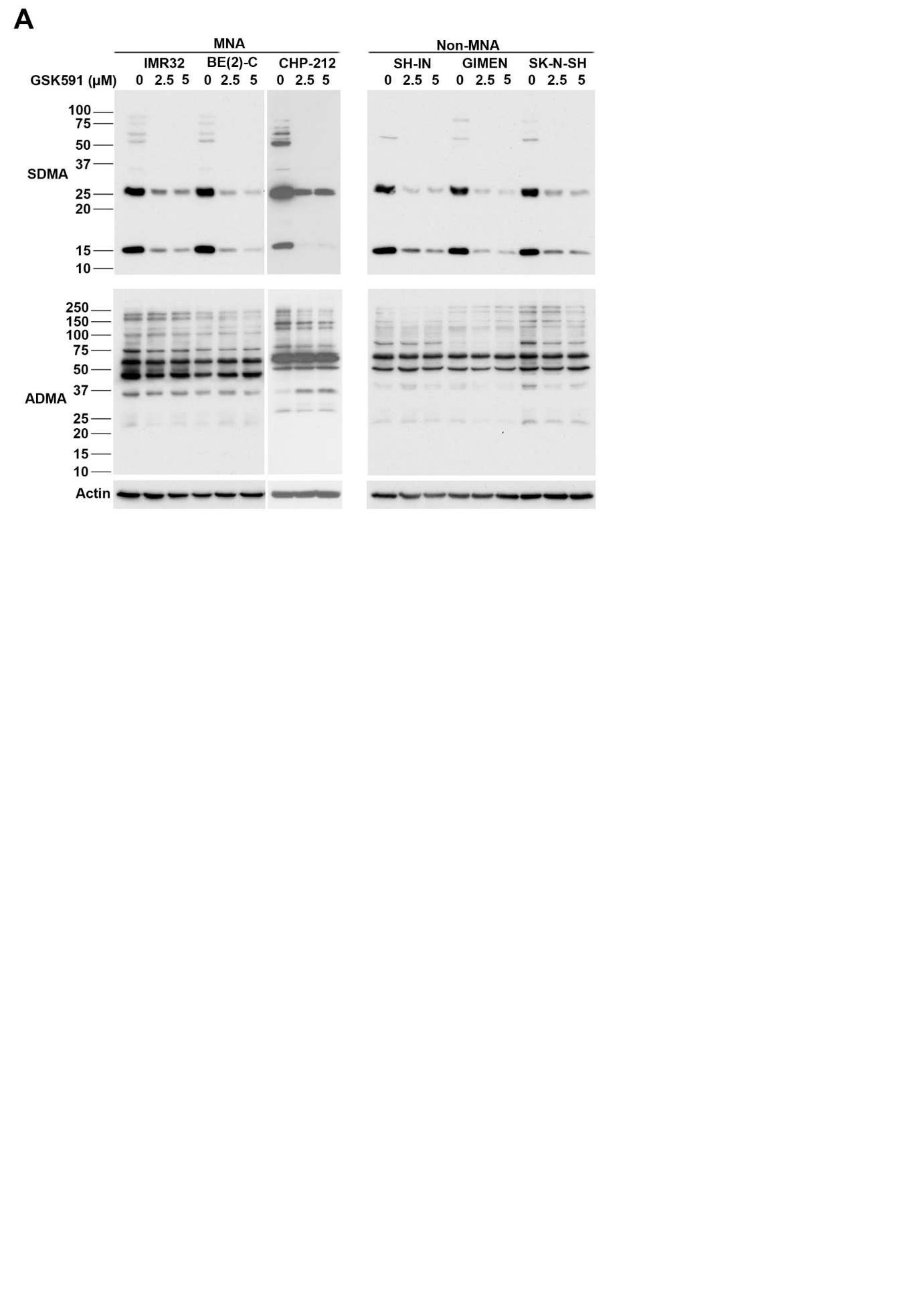


**SUPPLEMENTARY FIGURE 2**


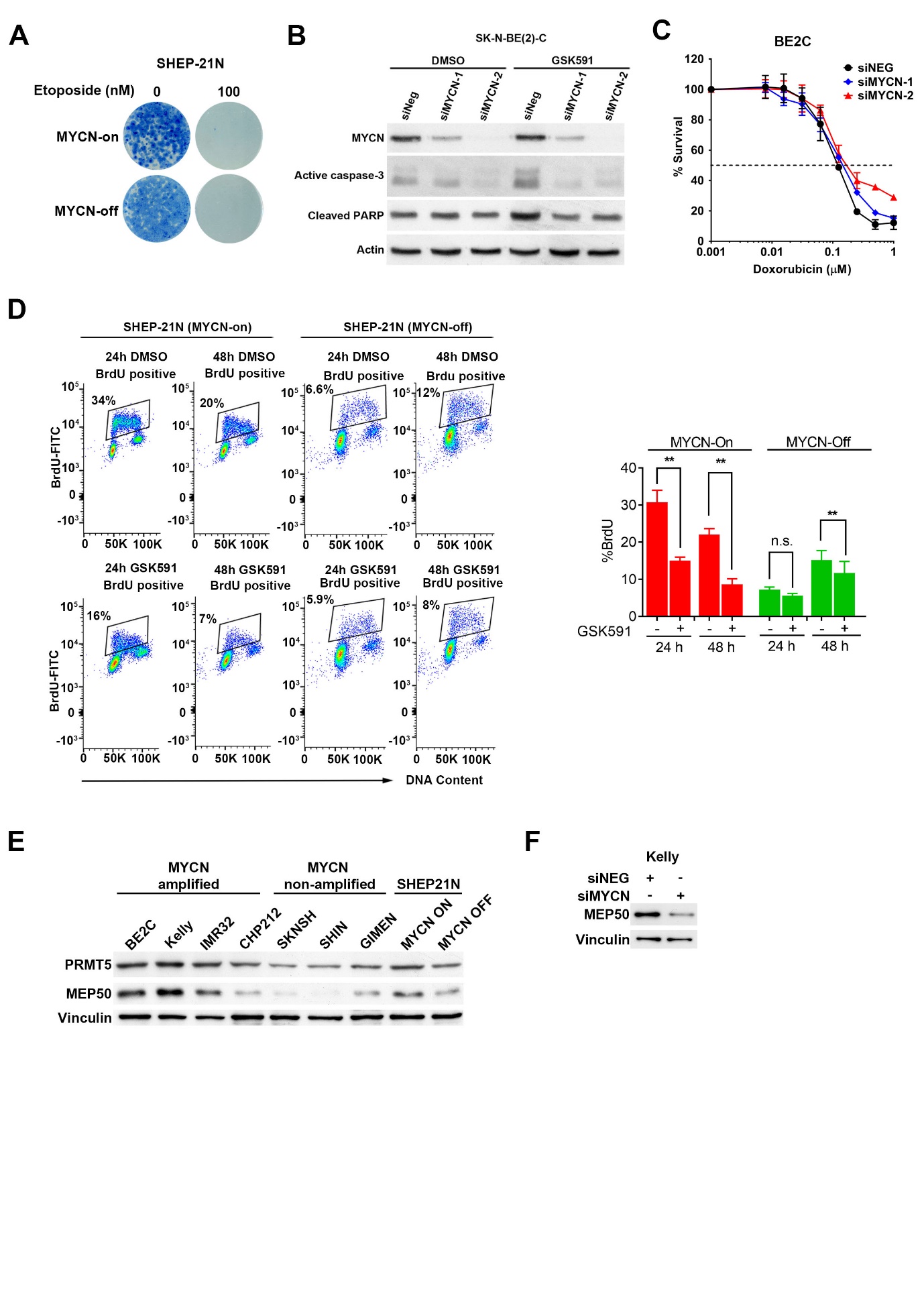


**SUPPLEMENTARY FIGURE 3**


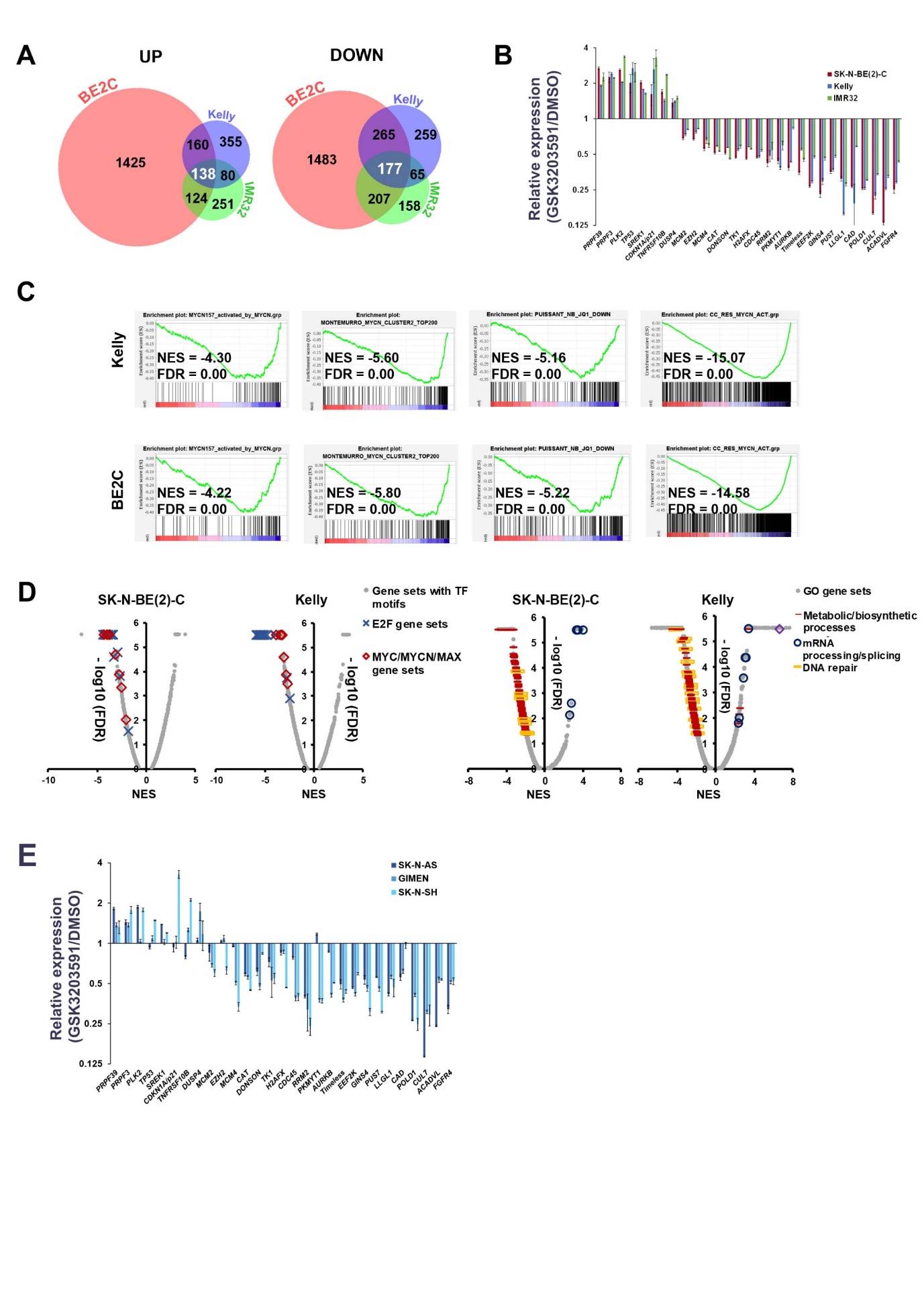


**SUPPLEMENTARY FIGURE 4**


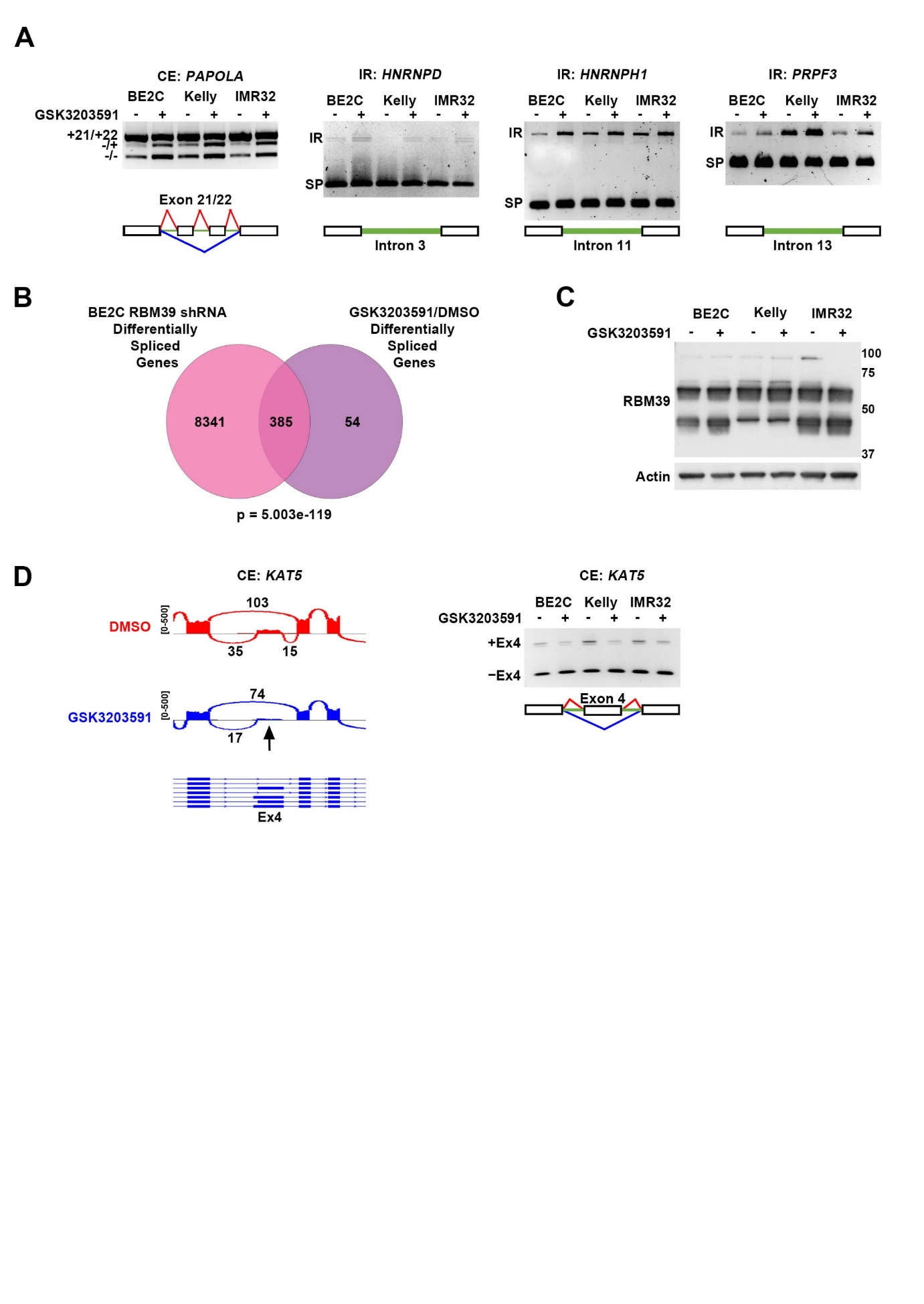


**SUPPLEMENTARY FIGURE 5**


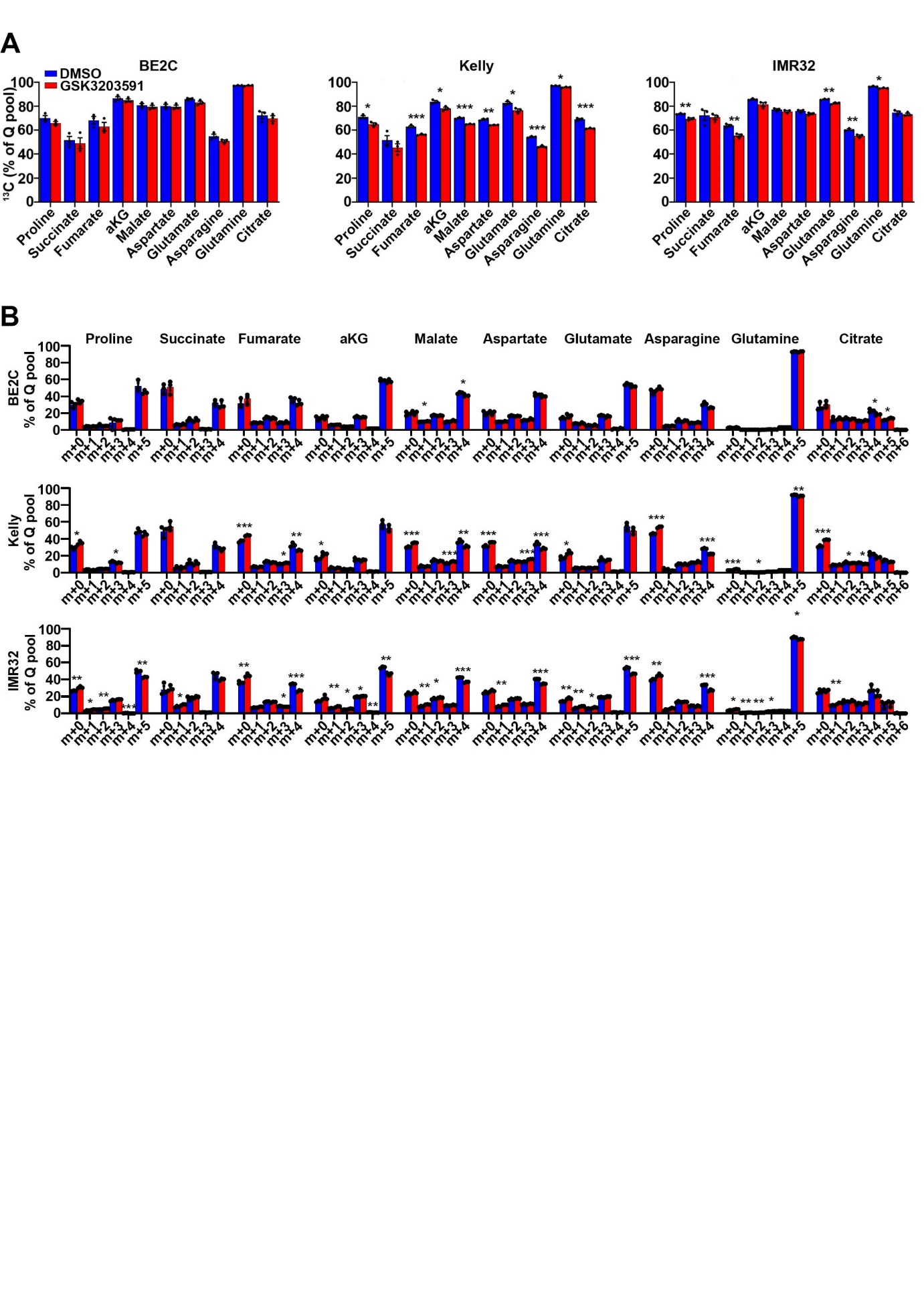


**SUPPLEMENTARY FIGURE 6**


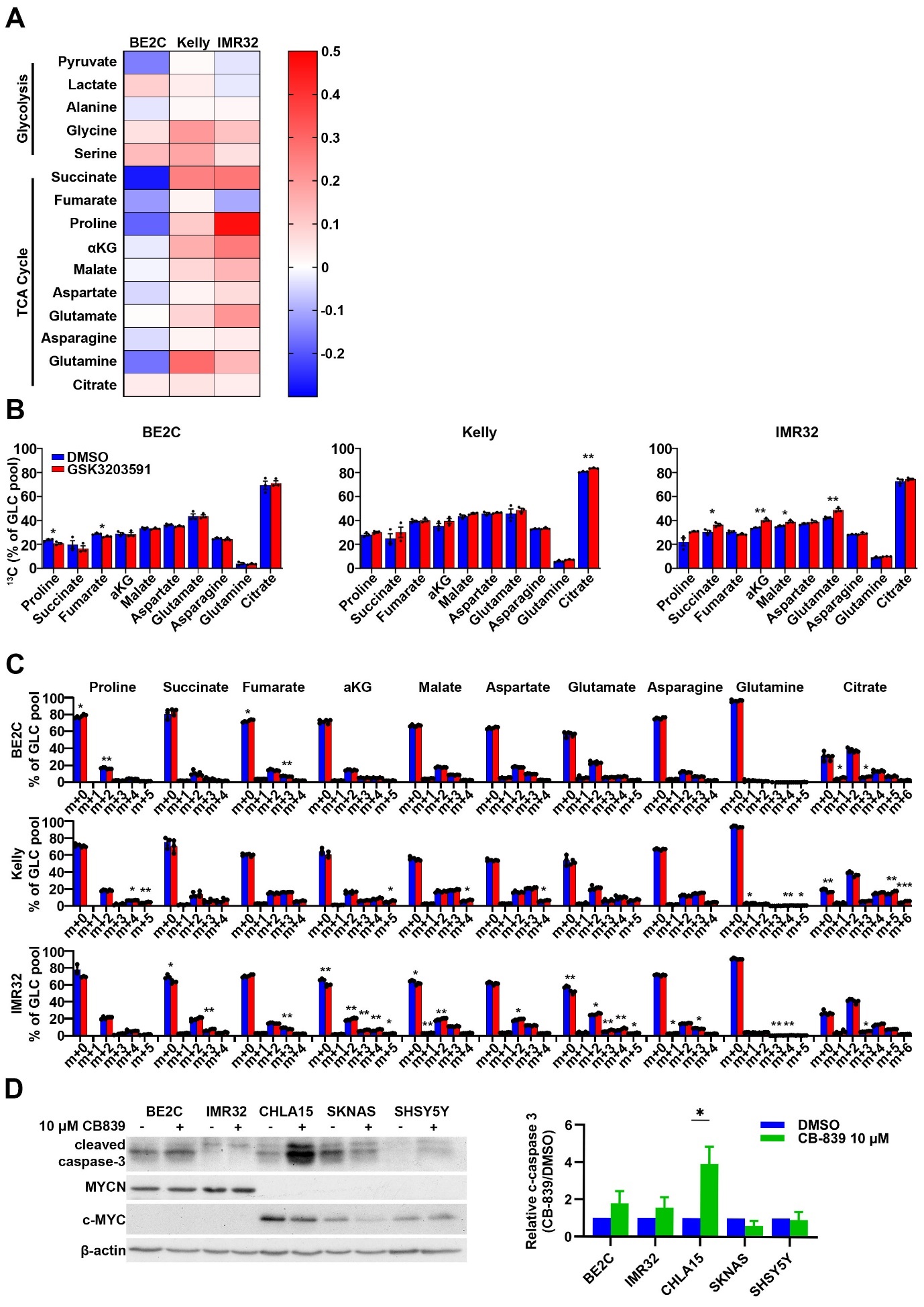


**SUPPLEMENTARY FIGURE 7**


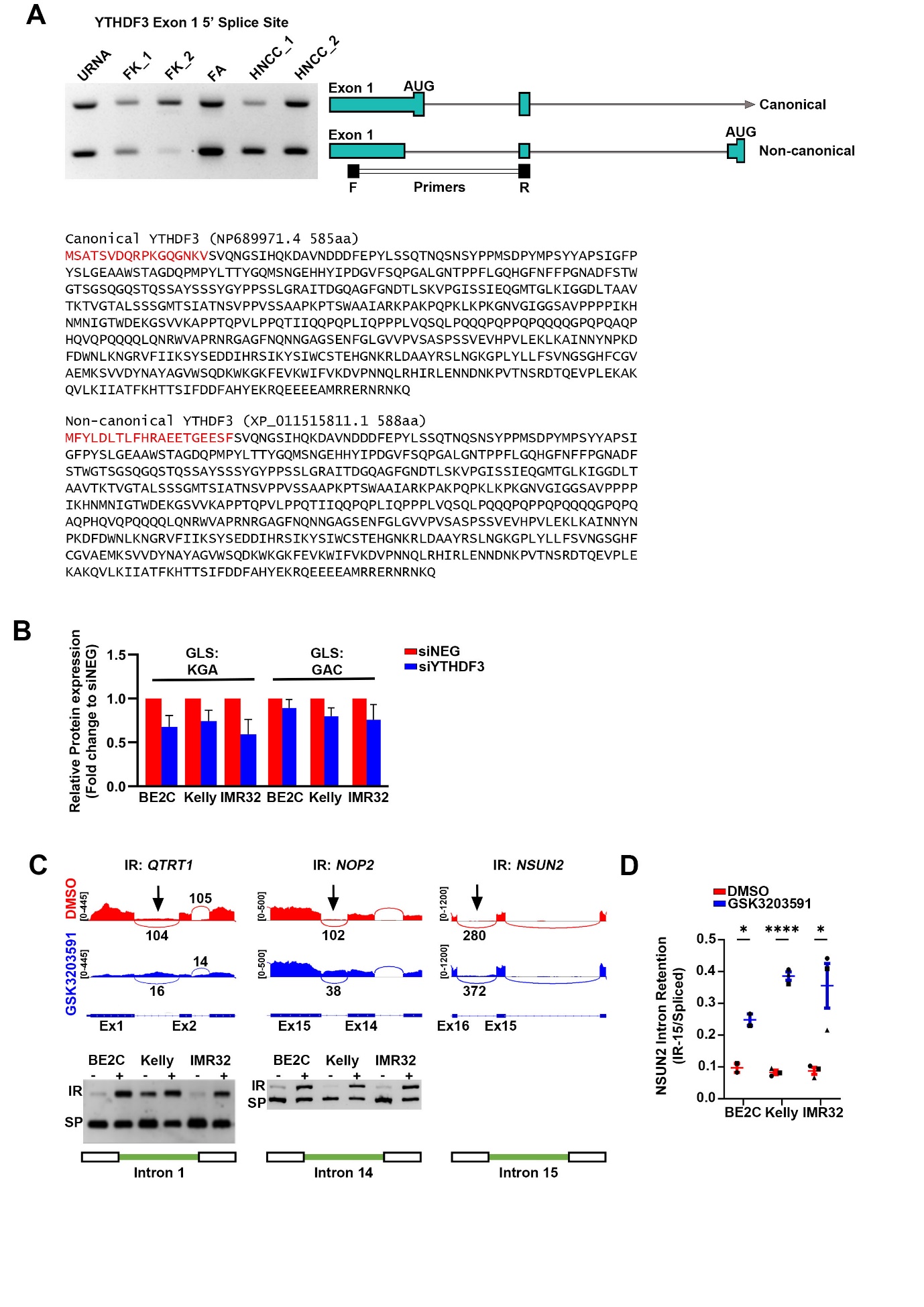

Supplement: Supplementary data [file EMS207915-supplement-Supplementary_data.zip › 1-s2.0-S030438352400658X-mmc3.docx]
